# Breaking barriers: The potential of nanosystems in antituberculosis therapy

**DOI:** 10.1016/j.bioactmat.2024.05.013

**Published:** 2024-05-17

**Authors:** Christian S. Carnero Canales, Jessica Ingrid Marquez Cazorla, Renzo Marianito Marquez Cazorla, Cesar Augusto Roque-Borda, Giulia Polinário, Rufo A. Figueroa Banda, Rafael Miguel Sábio, Marlus Chorilli, Hélder A. Santos, Fernando Rogério Pavan

**Affiliations:** a School of Pharmacy, Biochemistry and Biotechnology, Santa Maria Catholic University, Arequipa, 04013, Peru; b Tuberculosis Research Laboratory, School of Pharmaceutical Science, Sao Paulo State University (UNESP), Araraquara, 14800-903, Brazil; c School of Medicine, Santa Maria Catholic University, Arequipa, 04013, Peru; d School of Pharmaceutical Science, Sao Paulo State University (UNESP), Araraquara, 14800-903, Brazil; e Department of Biomaterials and Biomedical Technology, University Medical Center Groningen (UMCG), University of Groningen, Groningen, 9713 AV, the Netherlands; f Drug Research Program, Division of Pharmaceutical Chemistry and Technology, Faculty of Pharmacy, University of Helsinki, Helsinki, FI-00014, Finland

**Keywords:** Tuberculosis, *Mycobacterium tuberculosis*, Nanoscale drug delivery systems, Barriers, Drug resistance

## Abstract

Tuberculosis (TB), caused by *Mycobacterium tuberculosis*, continues to pose a significant threat to global health. The resilience of TB is amplified by a myriad of physical, biological, and biopharmaceutical barriers that challenge conventional therapeutic approaches. This review navigates the intricate landscape of TB treatment, from the stealth of latent infections and the strength of granuloma formations to the daunting specters of drug resistance and altered gene expression. Amidst these challenges, traditional therapies often fail, contending with inconsistent bioavailability, prolonged treatment regimens, and socioeconomic burdens. Nanoscale Drug Delivery Systems (NDDSs) emerge as a promising beacon, ready to overcome these barriers, offering better drug targeting and improved patient adherence. Through a critical approach, we evaluate a spectrum of nanosystems and their efficacy against MTB both *in vitro* and *in vivo*. This review advocates for the intensification of research in NDDSs, heralding their potential to reshape the contours of global TB treatment strategies.

## Highlights

• The granulomas may serve as sanctuaries for *Mycobacterium tuberculosis.*
• Current therapies hindered by malabsorption, low index, enzymatic degradation, and limited bioavailability.
• Only 45 % of the funding target proposed by the United Nations to combat tuberculosis in 2022 was secured.
• NDDS maximize therapy by sensitizing resistant strains and protecting against pH and enzymatic challenges.

## Graphical abstract

**Figure:**
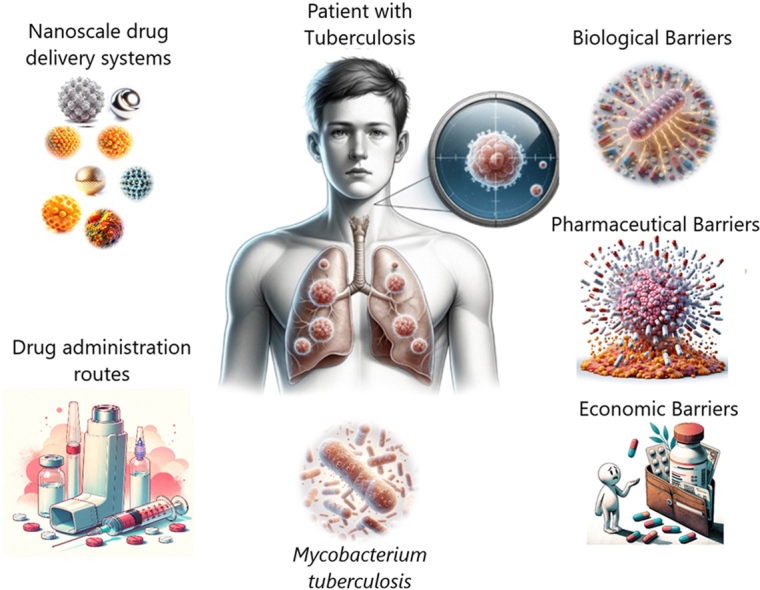

## 1 Introduction

Tuberculosis (TB) persists as one of the main threats to global health, being one of the most prevalent deadly infectious diseases caused by a single pathogen [1,2]. It is estimated that around the world, 10.6 million people developed TB in 2022. This increase represents an upward trend compared to previous years, where 10.3 million cases were recorded in 2021 and 10 million in 2020. COVID-19-related disruptions affected access to and provision of health services resulting in almost half a million extra TB deaths from 2020 to 2022 [3]. Additionally, the TB incidence rate experienced an increase of 3.9 % between 2020 and 2022, after having experienced an approximate decrease of 2 % annually between 2010 and 2020. This increase signals a significant change in the global landscape of TB [3]. This pathology presents a multitude of intricate challenges, encompassing biological, physical, and biopharmaceutical aspects [4].

TB is caused by the bacillus *Mycobacterium tuberculosis* (MTB), which was elucidated by Robert Koch in 1882 [5]. TB typically affects the lungs but can also affect other sites [6]. The common symptoms are coughing with sputum and sometimes bleeding, chest pain, weakness, weight loss, fever, and night sweats. These symptoms are mild for many months, leading to delays in seeking care that increase the risk of infection spreading to others [7].

The TB death rate is high without treatment. However, the current TB treatment lacks off efficacy due to the poor patient acceptance and completion of treatment attributed to its long duration and considerable side effects [8]. Obstacles to effective treatment include drug degradation before reaching the infectious site, limited solubility of pharmacological agents, drug interactions with medications used to treat comorbidities such as HIV/AIDS or diabetes mellitus, and significant side effects [9].

Isoniazid (INH) treatment requires regular monitoring due to the rare but dangerous risk of severe hepatotoxicity [10]. Rifampicin (RIF) exhibits low oral bioavailability due to low water solubility and stability of the active metabolite at acidic pH leading to repeated administration, which can cause dose-dependent toxicity and unwanted side effects [11]. Additionally, there is another significant factor in the eradication of TB, which is the growing resistance of *Mycobacterium tuberculosis* (MTB) to drugs used to treat the early stages of the disease [12,13]. Resistant TB can be classified into three groups: mono-resistant, poly-resistant, and multi-resistant (MDR), the latter being the most alarming, as it is resistant to the two first-line drugs, INH and RIF. There is also a fourth, rarer group, extremely drug-resistant TB (XDR-TB), resistant to all fluoroquinolones and at least one of the second-line drugs, such as amikacin, capreomycin, or kanamycin [14].

In 2020, 69 % of new TB cases worldwide were MDR, and 80 % of previously treated cases tested positive for MDR [15]. As with other microorganisms, the evolution of resistant TB is recognized as a natural biological event, resulting from a multifactorial scenario, infused by genetic alterations, poor clinical management, prolonged use of antimicrobial drugs, or poor adherence to TB treatment [16,17]. However, resistant strains require second-line drugs like aminoglycosides, polypeptides, fluoroquinolones, amikacin, kanamycin, thioamides, cycloserine, p-aminosalicylic acid and others, which present problems related to higher cost, greater toxicity, and greater side effects compared to first-line drugs [14,18,19].

In this scenario, nanotechnology emerges as a powerful tool due to its potential to significantly improve the therapeutic approach to TB. These advances encompass precise and controlled drug delivery at therapeutic doses, optimized macrophages uptake, maximizing the drug's bioavailability, improved distribution, decreased side effects, and an increase in patient adherence to treatment [20,21].

In this review, we report the current limitations of conventional TB therapies, including the physical, biological, biopharmaceutical, socio-economic barriers, as well as the inconsistent bioavailability, distribution, side effects and long-term therapies which contribute to low patients’ adherence. We also highlight the current nanomaterials-based approaches applied to overcome the obstacles of TB treatment including nanoemulsions, lipid-based nanoparticles, metallic nanoparticles, polymeric nanoparticles and mesoporous silica nanoparticles. Considering the few examples currently reported in literature, the future outlooks regarding the MDR-TB are also discussed in this review, aiming to promote the design of multifunctional and stimuli-responsive nanoplatforms that effectively tackle MTB diseases.

## 2 Limitations of conventional therapies in TB treatment

### 2.1 Biological barriers

#### 2.1.1 Nature of Mycobacterium tuberculosis

**Fig. 1:**
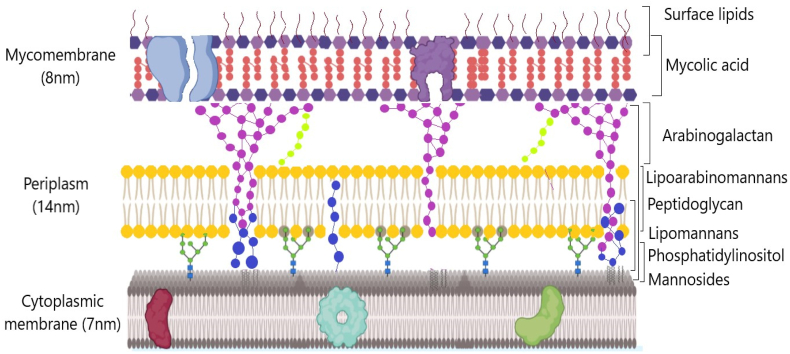
Structure of the mycobacterium cell wall. The innermost layer is the plasma membrane that acts as an impermeable barrier. Above it, the mAGP's peptidoglycan layer maintains cell integrity and increases pathogenicity. The mAGP's surface contains glycolipids linked by mycolic acids, which can function as virulence factors. This complex multilayered structure enables mycobacteria to resist antibiotic treatments and adapt to fluctuating environments. Created in biorender.com.

MTB is one of the strains of the MTB complex that causes TB in different types of hosts [22]. The mycobacteria of this group showed differentiation in terms of tropisms, phenotypes, and host pathogenicity, presenting a 99.9 % similarity at the nucleotide level and identical 16S rRNA sequences [23]. Despite the identification of the bacillus as the microorganism causing TB in 1882, Robert Koch revealed a mycobacterium with peculiar characteristics, different from those described and classified by Gram [24]. MTB was the first bacterium of the complex to have its genome fully sequenced in 1998. The researchers found out that the synthesis of the complex cell wall required more than 9 % of the metabolic demands on the genome's absorption capacity [25]. MTB's cell wall has shown a predominant architecture, organized in an intricate framework called mycolyl-arabinogalactan-peptidoglycan (mAGP) complex. This complex is notable for its inherent resistance to many conventional antibiotics, playing a critical role in the virulence of mycobacteria [23]. The mAGP's peptidoglycan layer exhibits critical peculiarities for maintaining cell wall integrity, regulating cell division, besides increases pathogenicity. These characteristics allow pathogens to survive within the host and resist pressures from antibiotic treatments [26]. The mAGP's outer surface hosts several glycolipids with sugar chains inserted along the cell wall, *e.g*., lipoarabinomannans, lipomannans, phosphatidylinositol mannosides, phenolic glycolipids, phthiocerol dimycocerosate, and acyltrehaloses. All of them are linked together through mycolic acids, showing potential to act as virulence factors and modulators of the host's immune system (Fig. 1) [27]. The cell coating of mycobacteria presents a multitude of layers of unusual complexity, facilitating its adaptability to fluctuating environments. The innermost layer of this envelope is the plasma membrane, which serves as the last line of defense and waterproofs the mycobacterium against external molecules [28].

MTB introduces a heightened challenge in TB therapy, stemming from its intrinsic ability to produce beta-lactamases [29]. Regarding these enzymes, Rv2068c (BlaC) stands out as a notable class A Ambler beta-lactamase due to its wide spectrum and resistance to clavulanate [30]. BlaC plays a pivotal role in MTB's defense against beta-lactam antibiotics, substantially undermining the effectiveness of prominent drugs like penicillins, imipenem, and cephalosporins [31,32]. However, carbapenems appear less affected by its influence. In a groundbreaking study, Kumar et al. [33] revealed that BlaC is not MTB's sole beta-lactamase activity source, which indicates that specific penicillin-binding proteins (PonA1, PonA2, DacB, DacB1, DacB2, PbpA, PbpB, Rv1367, Rv1730c, Rv2864c) might outperform BlaC in neutralizing beta-lactams.

Furthermore, MTB possesses sophisticated defense systems that bolster its survival odds. Prominent among these are efflux pumps—protein structures nestled in the cell membrane essential for both the bacterium's physiological functions and its antimicrobial resistance [34]. Coupled with the bacterial permeability barrier, these pumps decrease the influx of antimicrobial agents into the external cell membrane while aiding drug expulsion. Operating on the transmembrane electrochemical gradient of protons or sodium ions, these pumps actively dispel drugs from the cell's interior, negating their medicinal effects [35,36].

This expulsion mechanism has garnered interest, especially considering its association with antimicrobial resistance, encompassing primary TB drugs. Researchers have hypothesized that multiple efflux pumps, such as Rv1410c, Rv2936, and Rv0783, might induce a modest resistance to RIF [[37], [38], [39]]. Moreover, the Rv1258c efflux pump has been observed to offer RIF tolerance during macrophage infection [40]. Recent findings hint those mutations in Rv1258c within clinical strains could trigger pronounced resistance to medications like Pyrazinamide (PZA), streptomycin, and INH [41]. Alarmingly, resistance to Bedaquiline (BDQ), a drug recently launched to combat multidrug-resistant TB, have emerged shortly after its introduction. Within a year, increased resistance was attributed to the upregulation of efflux pumps like Rv0676c, Rv0677c, and Rv0678 [42,43]. These developments spotlight MTB's adaptability and intensify the intricacies of TB treatment, underscoring the pressing need for novel therapeutic avenues to confront escalating antimicrobial resistance.

#### 2.1.2 Latent tuberculosis and granuloma formation

MTB is a slow-growing bacterium that requires oxygen and organic compounds to multiply and obtain energy [44,45]. In cases of latent infection, the bacillus is in a state of rest, with limited oxygen, which favors the slow replication or even stops in replication without cells death [46]. It is estimated that a third of the world's population have been latently infected with MTB without any decrease in immunity [7]. The disease progression varies in each individual and the probability of contamination depends on some risk factors such as malnutrition, high alcohol consumption, smoking, and diseases, including HIV and diabetes mellitus [47].

MTB adopts a unique survival strategy by inhibiting the maturation of phagocytosis. The mycobacterium inhibits the acidification of the phagosome interior through the tyrosine phosphatase A protein and induces structural alterations, allowing macrophages to maintain a slightly neutral pH. This phagosomal acidification is reliant on the recruitment of the multi-subunit proton pump (V-ATPase) and allows MTB to persist in a relatively benign environment (pH 6.2) [48]. In addition, the invasion of macrophages by MTB stimulates the production of the granulocyte-macrophage colony-stimulating factor, which in turn initiates the expression of the cytokine-inducible SH2 domain-containing protein (CISH) through the mediation of STAT5 [49]. CISH can trigger ubiquitination and subsequent degradation of the A catalytic subunit of V-ATPase by proteasomes, in such a way that this microorganism blocks the recruitment of GTPases and V-ATPases to the phagosome membrane. It also has a secretion system (ESX-1) that damages and permeabilizes this membrane, allowing it to occasionally escape to the cytosol [50]. Parallelly, the nitric oxide synthase 2 (NOS2), induced by IFN-γ, activates the infected macrophages and inhibits the intracellular replication of MTB. LRG-47, a member of the 47 kDa guanosine triphosphate family, complements NOS2 and enhances macrophage resistance to infections [51]. During this procedure, infected macrophages accumulate lipid bodies, transforming into foamy macrophages, which play a crucial role in the establishment, conservation, and dissemination of granuloma infection [52].

**Fig. 2:**
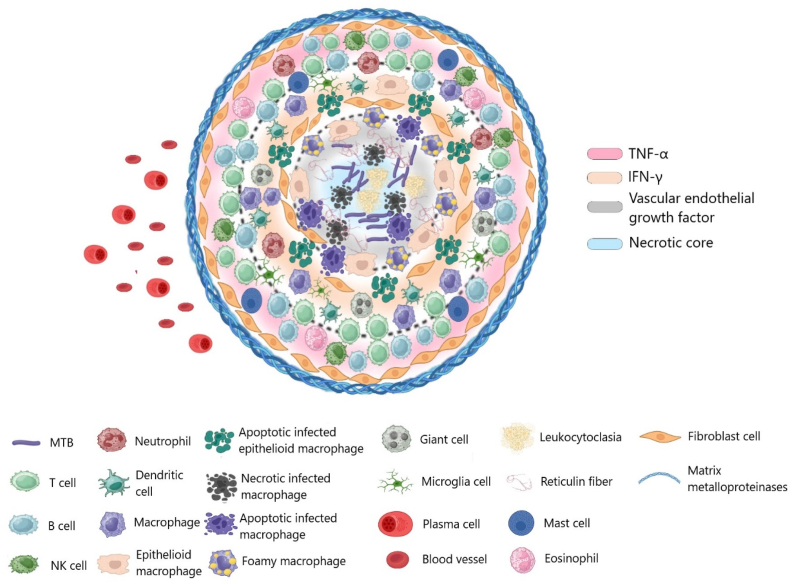
The dynamic, complex structure of TB granuloma encompasses cellular interconnections and distinct microenvironments. Created in biorender.com.

Granulomas are cellular aggregates produced in response to chronic inflammation. These structures contain a variety of immune cells such as monocyte-derived macrophages, foam cells, epithelioid cells, and multinucleated giant cells [53]. These immune cells orchestrate a defense response that activates T cells capable of inducing apoptosis in intracellular mycobacteria via enzymes like granzymes and granulysin [54,55]. Additionally, the release of cytokines and chemokines perpetuates inflammation by recruiting other immune players, including B cells and natural killer (NK) cells. This response is characterized by the production of inflammatory mediators like interferon-gamma (IFN-γ) and tumor necrosis factor-alpha (TNF-α). In this environment, naïve CD4^+^ cells, once stimulated by interleukin-12 (IL-12), differentiate into T helper 1 (TH1) cells, furthering the immune response (Fig. 2) [[56], [57], [58]].

However, recent research has shown that granulomas, previously considered sites of MTB growth inhibition, may act as sanctuaries for mycobacteria. It has been proposed that granulomas present a more dynamic and intricate structure than originally believed, characterized by interrelated areas with diverse microenvironments [59]. Additionally, a recent study conducted in zebrafish infected with *Mycobacterium marinum* showed that monocyte recruitment in the granuloma can favor pathogen proliferation [60]. Therefore, it is of great importance to optimize and design target drug delivery systems towards macrophages or the inner of the granuloma.

#### 2.1.3 Drug resistance and altered gene expression

For two decades, many MTB infection cases have presented variants that developed mechanisms of resistance to existing drugs, due to lack of adherence to therapy [61]. The WHO's “Global Tuberculosis Report 2023” reveals that the estimated annual number of MDR-TB cases worldwide in 2022 remained steady compared to 2020 and 2021, marking a concerning tally of 410,000 affected individuals (World Health Organization, 2023).

Unlike other infectious diseases, there are different types of TB resistance, and they are classified according to the applied drugs in therapy. When the patient is infected with bacteria resistant only to RIF or INH, it is classified as monoresistant. If it is resistant to both drugs (RIF and INH), it is called MDR. Additional resistance to fluoroquinolones, BDQ, or Linezolid (LNZ) are classified like XDR [62]. The mycobacteria's cell wall functions as a physical barrier to drug permeability, participating in sustaining pathogenicity, however, when resistance is presented, *e*.*g*., in XDR cases, the basal layer of peptidoglycans becomes denser and almost fused with the electron-transparent layer [63].

Nguyen et al. [61] identified over 25 mutated genes responsible for encoding 27 proteins, which in turn confer resistance to 10 current drugs. Recently, researchers have found out 34 genes linked to resistance against 17 drugs, as listed in Table 1. While other genes have been noted as potential markers for drug resistance, the WHO analysis excluded them due to a lack of association with resistance, based on their methodology [64,65].

**Table 1:** MTB genes with resistance mutations against various drugs, the mode of action and genes product. Data Source: Google Scholar and PubMed, utilizing the search terms “*Mycobacterium tuberculosis*” or “tuberculosis” AND “mutation” or “resistance” or “resistance gene” AND the name of each drug used in TB therapy (*e.g.*, “Rifampicin”).

Drug	Related Gene	Gene Product	Mode of Action	Reference
Rifampicin	*rpoB*	β subunit of RNA polymerase	Inhibiting transcription	[66]
Isoniazid	*katG*	Catalase/peroxidase	Inhibiting mycolic acid biosynthesis and other metabolic processes	[67]
	*inhA*	Enoyl reductase		[68]
	*ahpC*	Alkyl hydroperoxide reductase		[68]
	*Ndh*	NADH dehydrogenase II		[69]
Pyrazinamide	*pncA*	Pyrazinamidase/nicotinamidase	Inhibiting *trans*-translation	[70]
	*rspA*	Ribosomal protein S1		[71]
	*glpK*	Glycerol-3-kinase		[72]
Ethambutol	*embCAB*	Arabinosyl transferase	Inhibiting arabinogalactan synthesis	[73]
	*embB*			[74]
Levofloxacin/Moxifloxacin	*gyrA/gyrB*	DNA girase	Inhibiting DNA girase	[75,76]
Capreomycin	*Rrs*	16S Rrna	Inhibiting protein synthesis	[77]
	*tlyA*	rRNA methyltransferase		
Amikacin/Kanamycin	*Eis*	Aminoglycoside acetyltransferase	Inhibiting protein synthesis	[78]
	*whiB7*	MDR transcription regulator		[79]
	*Rrs*	16S Rrna		[80]
Streptomycin	*rpsL*	S12 ribosomal protein	Inhibiting protein synthesis	[81,82]
	*whiB7*	MDR transcription regulator		[83]
	*Rrs*	16S Rrna		[84]
	*gidB*	7-methylguanosine methyltransferase		[85]
Ethionamide	*ethA*	Flavin monooxygenase	Inhibiting mycolate biosynthesis	[86]
	*ethR*	ethA transcriptional repressor		[87]
	*Ndh*	NADH dehydrogenase II		[88]
	*mshA*	Glycosyltransferase		[89]
	*inhA*	Enoyl reductase		[90]
d-Cycloserine	*alrA*	d-Alanine racemase	Inhibiting peptidoglycan synthesis	[91]
	*cycA*	d-Alanine-d-alanine ligase		
	*Ddl*	Amino acid transporter		
p-Aminosalicylic acid	*thyA*	Thymidylate synthase A	Inhibiting folate biosynthesis	[92]
	*dfrA*	Dihydrofolate reductase		
	*folC*	Dihydrofolate synthase		[83]
	*ribD*	Dihydrofolate reductase analog		
Bedaquiline and Clofamizine	*atpE*	ATP synthase	Inhibiting ATP synthase	[93]
	*Rv0678*			[94]
Linezolid	*rpLc*	C154R on L3 ribosomal protein	Inhibiting protein synthesis	[95]
Delamanid	*fbiA, fbiB, fbiC and fgd1*	Coenzyme F420	Inhibiting mycolic acid biosynthesis	[96]

MTB presents five other forms of intrinsic resistance, which are: target alteration, where a bacterium modifies the structure of the drug's binding target; modified drug, where bacteria can add specific groups to the drug molecule; drug degradation, when mycobacteria manage to break down the active molecule; drug efflux, when a bacterium can excrete the drug that has entered its cytoplasm; and target mimicry, a specific case against fluoroquinolones, where a bacterium manages to neutralize the drug action using a protein to mimic the DNA structure [97]. The evolution of drug resistance caused by extrinsic and intrinsic factors leads not only to adverse drug responses, but also enables MTB to survive within the host's main immune response feature, the granuloma, evolving towards a much more complex structure than the simple innate inflammatory response, making it more than a simple physical barrier [98].

The differentially expressed genes (DEG) appear during the active TB development. Chen et al. [99] described the 5 main genes that can change their expression due to the disease development, being the key genes of TB: STAT1, GBP5, OAS1, CTNNB1, and GBP1. The importance of understanding genetic expression and polymorphisms lies on their relationship with sensitivity, clinical characteristics, and adverse effects of anti-TB drugs in patients [100]. One of the most common adverse effects of anti-TB drugs is liver injury (ATDILI), which is associated with antioxidant enzymes, with NOS2 and MAFK genes, which is related to ATDILI sensitivity in patients [101]. The polymorphisms rs9906835, rs944725, and rs3794764 of the NOS2 gene show higher risk of suffering from ATDILI, while the polymorphism rs3735656 of the MAFK gene was significantly associated with decreased risk of ATDILI [102].

The first-line drugs are involved in lipid and purine metabolism; therefore, they are implicated in the toxicity responsible for liver injury [103]. Among first-line anti-TB drugs, PZA is the main drug causing drug-induced liver injury (DILI), developing injury at the hepatocellular level. PZA-related DILI could be of late onset and prolonged, and the slow acetylator phenotype of NAT2 is a possible risk factor [104]. These results are related to the retrospective investigation performed by Shu et al. [105] concluded that the relationship of DILI with PZA was 3.7 patients per month; being the most common compared to INH or RMP.

Moreover, data presented by G. Zhang et al. [106] showed that individual intracellular bile acids increase after INH treatment, and the accumulation of bile acids in the organism can become toxic regardless the INH concentration, which causes liver damage through various mechanisms (mitochondrial dysfunction, endoplasmic reticulum (ER) stress, and inflammatory response), besides to observe necrosis and hepatic steatosis at concentrations of 180, 300, and 600 mg/kg. However, liver damage was not detected in rats treated with 60 mg/kg. These results were confirmed by Hassan et al. [107] showed that the hepatic necrosis and steatosis in animals exposed to high INH doses are related to mitochondrial dysfunction, oxidative stress, and alteration of bile acids homeostasis.

RIF is associated with hepatocyte toxicity primarily through its induction of ER stress via multiple mechanisms. Firstly, RIF's linkage to bile acids accumulation promotes an increase in intracellular calcium and oxygen stress, pivotal in driving ER stress. Secondly, RIF indirectly triggers ER stress by activating the pregnancy X receptor (PXR), which in turn enhances the expression of CYP enzymes. Abnormal levels of CYP enzymes can lead to ER stress through the activation of the X-box binding protein 1 (XBP1) via the inositol-requiring enzyme 1 (IRE1) signaling pathway, ultimately resulting in cell apoptosis. This sequence of events outlines the PXR-CYP-XBP1-IRE1 pathway as a critical mechanism of ER stress [108,109]. In the study carried out by Zhang & Yew [110], it was confirmed that RIF-resistant TB was associated with the RNA polymerase β subunit gene (rpoB) mutation, corresponding to approximately 96 % of the cases.

The importance of PZA in the treatment of MDR-TB is recognized, however, more and more cases of resistance have been described worldwide [111]. As a drug, PZA needs to be converted to its active form, pyrazinoic acid (POA), which is accomplished through the action of the enzyme pyrazinamidase (PZase) that is encoded by the pncA gene and contains 561 nucleotides. Loss of PZase activity results in mutations in the pncA gene, which is the main mechanism of resistance to PZA [112]. The most frequent pncA gene mutation is located at codon 132 and results in the substitution of the amino acid glycine (Gly) to aspartic acid (Asp). This statement was confirmed in the investigation conducted by Pang et al. [113], showing that 88 % of PZA-resistant strains had a mutation in the pncA gene. Therefore, pncA gene analysis reveals precise information about PZA sensitivity. The glpK gene has also been identified in the resistance to anti-TB drugs, and especially PZA, being responsible for encoding the enzyme glycerol-3-kinase, necessary for the catabolism of glycerol.

In the study conducted by Bellerose et al. [72] the *in vitro* growth of MTB in glycerol increased drug sensitivity and the bacteria deficient in the glpK gene persisted during *in vivo* treatment, especially in regimens with PZA. This result showed that changes or mutations in the glpK gene are significantly associated with antibiotic resistance. It was also identified that the genes glgA and glgC from the glycogen synthetic pathway increase antibiotic activity.

The association of RIF and INH treatments leads to the accumulation of endogenous protoporphyrin IX in the liver through the heme group biosynthetic pathway. Alanine synthase 1 and ferrochelatase are the key enzymes regulating heme production. Zhang et al. [114] identified that the polymorphism rs11660001 in ferrochelatase in women is related to the development of ATDILI. Anti-TB drugs generally target constantly replicating bacteria, so a state of latency is a delay in the fight against TB.

In addition, supplementation with vitamin C could help to eliminate MTB. Sikri et al. [115] demonstrated that vitamin C associated with Anti-TB drugs such as RIF, INH and PZA manages to eliminate both latent and replicating bacterial subpopulations, proving that vitamin C can be an interesting adjuvant in TB treatment.

### 2.2 Biopharmaceutical barriers

#### 2.2.1 Malabsorption of drugs

The TB treatment from a clinical administration standpoint poses significant challenges, especially given the bioavailability issues associated with oral and intravenous drug delivery [[116], [117], [118]]. Although the oral administration route is the most widely used and practical method, its effectiveness is largely determined by the drug's ability to be absorbed through the intestinal wall [119]. Drug permeability across this selection barrier is crucial, and it is mainly impeded by the enteric epithelium and enzymatic degradation. Given that roughly 90 % of absorption takes place in the small intestine, it is evident that malabsorption (MA) challenges predominantly arise there. Additionally, hepatic metabolism can further diminish the drug's ultimate bioavailability [120].

The absorption and plasma availability of intravenously administered drugs are influenced by several factors, including a patient's hydration level, the volume of fluids administered, and the amount and type of plasma and tissue proteins. These elements can change the volume of distribution, leading to fluctuations in plasma concentrations and, consequently, the drug's effective duration [121].

As with other antibiotics used for other infectious conditions, the oral MA of anti-TB drugs constitutes a cause, albeit rare, but predisposing to treatment failure in TB's patients. In those patients receiving anti-TB therapy, whose condition does not show clinical, microbiological, or radiological improvement, oral MA should be considered as one of the possible causes [122]. It is considered that the MA of one or more drugs leads to treatment failure, the development of adverse clinical events, disease progression, and possible resistance to the drugs [123].

Most patients diagnosed with TB, in the absence of comorbidities, absorb the drugs effectively [124]. However, certain systemic conditions related to the digestive system can cause MA. Notably, cholestasis, liver disease, inflammatory bowel and pancreatic diseases, gastritis, hypothyroidism, amyloidosis, alcoholism, and diabetes are among the predominant clinical conditions leading to MA syndrome [125]. These conditions can reduce the serum concentrations of anti-TB drugs, resulting in therapeutic failure [126]. Patients with enteropathies have reduced anti-TB drugs absorption, which is particularly problematic in the case of RIF since its absorption depends on the permeability and solubility of the gastrointestinal tract (GIT) that is affected by pH and intestinal transit [127].

Another factor to consider in the drugs MA is the intake of food with the anti-TB drugs administration. Saktiawati et al. [128] concluded that the consumption of food with anti-TB drugs decreases the maximum concentration and bioavailability of RIF and INH by 22 and 42 %, respectively, with non-significant effect for PZA (10 %). Compared to anti-TB drugs taken on an empty stomach, this influence could eventually affect the response to the treatment and increases the risk of resistance. These results are in line with Lin et al. [129], who measured the absolute bioavailability for anti-TB drugs post-feeding and fasting. The absolute bioavailability values for RIF, INH, and ethambutol (EMB) were 87, 93, and 87 % after feeding, and 71, 78, and 82 % during fasting, respectively. Another factor of consideration is the use of antacids usually indicated in those patients who present adverse effects to the therapeutic regimen including nausea, vomiting, and abdominal pain, which are common during the first weeks of treatment. In the study developed by Lin et al. [130], the authors evidenced that antacids reduced the maximum concentration of EMB although it does not significantly affect INH, RIF, and PZA.

**Fig. 3:**
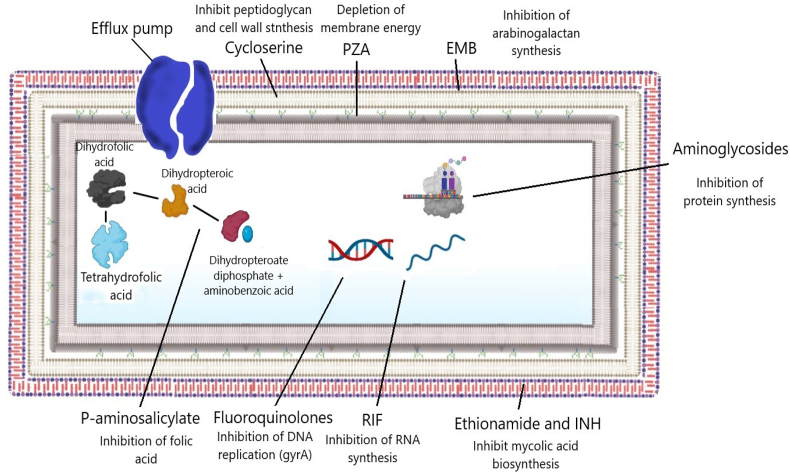
Mechanism of action of the main drugs used in TB therapy. Created in biorender.com.

The mechanism of anti-TB drug action is different in each one, *e.g*., RIF acts at the level of the RNA polymerase preventing the transcription of DNA to RNA [131], whereas the INH needs to be activated by the enzyme KatG, inhibiting the enzymes related to the synthesis of cell wall lipids, acting mainly on the inhA protein in charge of the cell wall synthesis [132]. The PZA requires prior activation in acidic pH, and its active form, pyrazinoic acid, show bactericidal action [133,134]. The EMB also acts at the level of the cell wall, preventing the synthesis of arabinolactan by blocking its polymerization by inhibiting the binosyltransferase [135], and the fluoroquinolones are going to inhibit the enzyme topoisomerase II, known as DNA gyrase (Fig. 3) [136].

#### 2.2.2 Interaction with other drugs

The synergistic interaction of anti-TB drugs is well-known. Each drug's unique mechanism of action enables it to operate independently, which could lead to competition with, or even neutralization of the bactericidal effects of other drugs [137]. Moreover, when co-administered optimally, these drugs can produce pharmacological synergy. As such, meticulous attention is required when prescribing these drugs either singly or in combination to prevent adverse clinical outcomes [138]. RIF is a lipophilic drug available in both oral and intravenous forms, which presents half-life ranges from 2 to 5 h [139]. RIF acts as an inducer for various cytochrome oxidases P450 enzymes, including CYP3A4, CYP2A, CYP2B, CYP2C, and CYP3A. This interaction can lead to a plethora of side effects and reduces the efficacy of concurrently administered drugs [140].

INH administered via oral, intramuscular, and intravenous formulations inhibit cytochrome oxidase P450 enzymes and monoamine oxidase [141]. A critical interaction during TB treatment is its combination with RIF, which can lead to hepatotoxicity [142]. The risk of liver damage escalates when these drugs are combined, due to their additive effects. Several other factors heighten the toxicity risk, including the concurrent use of drugs such as acetaminophen, oral antidiabetics, and ketoconazole [143]. Alcohol consumption, advanced age, and malnutrition also elevate the risk of liver damage, neuropsychiatric disorders, and hematological abnormalities [144]. These conditions are often linked to oxidative stress. On the other hand, PZA shows synergistic activity with RIF, which has good oral absorption unlike other anti-TB drugs. The mechanism of action is based on the inhibition of Coenzyme A synthesis by binding to the enzyme aspartate decarboxylase, blocking this important enzymatic pathway of MTB [145].

EMB oral and intravenous formulations work by targeting and inhibiting the enzyme arabinosyltransferase, a key player in the synthesis of the MTB cell wall. Meanwhile, Levofloxacin and Moxifloxacin stand out as the top-recommended fluoroquinolones for addressing MDR-TB, operating by curtailing the activity of the MTB DNA gyrase [146]. It is worth noting that the co-administration of these anti-TB drugs with substances such as sucralfate, iron salts, zinc salts, or antacids enriched with magnesium or aluminum can markedly diminish their absorption and overall bioavailability [147].

#### 2.2.3 Comorbidities

TB co-infected patients with other comorbidities like HIV, Diabetes, influenza, malaria, hepatitis, and parasitosis, are usually administered with antibiotic, antifungal, retroviral, and antiviral drugs, presenting in most cases drug interaction problems [148]. HIV infection remains a substantial challenge for global public health, compounded by tuberculosis as a significant comorbidity. In 2022, it was estimated that about 78,000 men, 58,000 women, and 31,000 children succumbed to TB/HIV co-infection [3]. Alarmingly, around 2.1 % of patients undergoing combined treatment for both conditions experienced such severe toxicity, confirming that it is essential to modify their treatment regimen [149].

TB/HIV co-infected patients also have a higher risk of developing MDR-TB [150]. This endemic comorbidity is very common in Africa where the progression is rapid for HIV infection [151]. These patients predispose to anti-TB drugs MA especially RIF and INH [152]. Agents used for the MDR-TB treatment demonstrate simultaneous enzyme inhibition and/or induction that may significantly influence the outcomes of antiretroviral therapy [153]. It has been documented that at least five drugs, forming the crux of the commonly administered regimen for MDR-TB, inhibit the key metabolic pathways of antiretrovirals, encompassing CYP3A4, CYP2B6, CYP2C9, and CYP2D6 [154]. As a result of enzymatic induction, RIF alters the concentrations of dolutegravir (DTG), necessitating a dual DTG dosage during contraindication of the drug-sensitive TB treatment in HIV patients. This interaction risk could potentially be exacerbated by escalating RIF dosages [155]. In another study conducted in coinfected HIV and TB patients undergoing treatment, it was evidenced that the decrease in serum concentrations of dolutegravir led to an adjustment of the daily drug dose to maintain its effectiveness in addition to increasing the risk of hepatic toxicity [156].

Moreover, diabetes mellitus (DM) has emerged as a highly prevalent disease globally [157]. At the same time, it has been observed that the interaction of this pathology with TB can lead to particularly devastating consequences for public health. The individuals with DM who contract TB have a high probability of developing MDR-TB [158], besides experience a high rate of therapeutic failure [159].

This complex relationship between DM and TB is detailed by Liu et al. [160]. The authors explained how the comorbidity of these two conditions can lead to the most severe adverse effects. In a significant number of cases, the severity of these complications leads to treatment interruption, which in turn endangers the patient's life and promotes the emergence of MDR-TB. From an economic perspective, this comorbidity represents a considerable burden. Treatment costs are increased due to the need for stronger drugs and the demand for more intensive healthcare services. Thus, the challenge of treating TB in patients with DM is of great magnitude from a clinical and financial perspective. DM also causes a change in the absorption of medications, damages liver and kidney function leading to nephrotoxicity and hypothyroidism, the interaction alteration is mutual, as TB treatment can worsen the glycemic control of patients [161].

In the study carried out by Soedarsono et al. [162], the authors concluded that TB treatment in patients with DM is associated with auditory damage, in a relationship of 26.8 %. This is confirmed by Sharma et al. [163] demonstrated the ototoxicity related to DM and hypertension. Moreover, anti-TB drugs are related to eosinophilia (17.8 %) and skin reactions (61.5 %) in addition to abnormalities in liver function, showing that this adverse effect is related in turn to patients presenting eosinophilia [164].

#### 2.2.4 Bioavailability and distribution

The field of biomedicine is evolving towards precise treatments, emphasizing personalized therapeutic interventions tailored to a patient's sensitivity to specific drugs. In anti-TB therapy, new guidelines advocate for strategies based on drug sensitivity [165]. In parallel, therapeutic drug monitoring (TDM) has emerged as an essential methodology, allowing the quantification of post-administration anti-TB drug, which facilitates dosing adjust, minimizes adverse effects and drug interactions, and optimizes therapeutic response while preventing the development of MDR-TB [166].

To establish the typical plasma concentrations of anti-TB drugs, studies suggest that peak concentrations are attained roughly 2 h after administration. Measurements taken 6 h post-administration can shed light on delayed absorption or hastened metabolism in patient [167]. Nevertheless, while TDM is invaluable for adjusting doses and preempting drug interactions, it does not capture every variable that might influence treatment outcomes. Thus, it is essential to use TDM in conjunction with assessments of clinical, bacteriological, and radiological data [167,168].

From a pharmacodynamic perspective, genetic profiling of patients with pulmonary TB is a promising method to predict treatment success. The NAT2 gene plays a crucial role in the pharmacokinetics of anti-TB drugs, encoding the liver enzyme N-acetyltransferase-2. This enzyme is responsible for metabolizing drugs, affecting their active concentrations in the plasma. Variations in the NAT2 gene lead to three acetylator phenotypes: fast, intermediate, and slow. Slow acetylators are associated with adverse reactions such as nausea, drug-induced liver injury, peripheral neuropathy, and sideroblastic anemia, potentially necessitating discontinuation of treatment [169]. In contrast, fast acetylators may metabolize drugs too rapidly, resulting in subtherapeutic plasma concentrations and increasing the risk of treatment failure and resistance [170]. Fast acetylators are twice as likely to suffer microbiological treatment failures as slow acetylators [171]. INH displays high hydrophilicity and low stability at physiological pH values, with limited membrane permeability, requiring higher doses to be effective [172]. RIF is poorly soluble in water but increased solubility in acidic conditions [173].

In this context, to overcome these pharmacological barriers, the potential of pulmonary administration has been explored. This method offers increased solubility, reduced doses, enhanced stability, fewer side effects, and improved therapeutic outcomes [174]. Building on this, Patil et al. [175] aimed to increase the solubility of delamanid, given its inherently low solubility. The main goal was to enhance solubility by forming a complex with cyclodextrin. Among various cyclodextrins, 2-hydroxypropyl-β-cyclodextrin (HP-β-CD) was the most effective, increasing solubility by 54-fold. In comparison, Sulfobutylether-β-cyclodextrin (SBE-β-CD) and Alpha Cyclodextrin Derivative (HP-ɣ-CD) increased solubility by 27-fold and 13-fold, respectively. Successful complexation of delamanid with HP-β-CD reduced the minimum inhibitory concentration by up to 4 times compared to free delamanid and maintained 90 % stability for up to 4 weeks [176].

#### 2.2.5 Long-term therapy and adverse effects

TB treatment differs from other bacterial infections treatment due to the limited and evolving understanding of its clinical pathogenic spectrum. This understanding is influenced by the continuous metabolic activity of the bacteria and the antagonistic immune responses it elicits. MTB has a prolonged generation time and the ability to enter periods of latency with limited metabolic activity, which hinders the action of antimicrobials [177].

Given the metabolic diversity among MTB bacilli populations, treatment strategies for TB need extended periods and the combination of different pharmaceutical agents [178]. This approach is informed by genetic research, which attributes the resistance of MTB to monotherapy to spontaneous genetic mutations affecting the enzymes or genes involved in drug activation. MDR arises from the sequential accumulation of mutations across multiple loci of independent genes [179]. The therapeutic challenges are further complicated by the diverse microenvironments that MTB can inhabit, including pulmonary cavities, empyema pus, or solid caseous material. These locales present barriers to antibiotic penetration and often exhibit low pH levels, which can inhibit the activity of most antibiotics [180]. Consequently, the clinical manifestations of active TB disease vary significantly in severity and presentation, ranging from pulmonary to extrapulmonary forms [181].

The current therapy for TB treatment is based on the combination of 4 drugs for a minimum period of 6 months. In the first 2 months, INH, RIF, PZA, and EMB are administered, and for the following 4 months only RIF and INH are administered [182]. TB treatment consists of 2 phases, the intensive phase is the highest load of bacilli and there is a greater risk of generating resistance, which justify the use of several associated drugs in this phase. In the continuation phase, the treatment is prolonged using drugs with the greatest sterilizing effect that curtails metabolic activity since this causes relapses in treatment, being that the RIF stands out as the essential drug with the greatest sterilizing effect [183].

Each anti-TB agent plays a vital role in the treatment regimen. INH, integral to the initial stages of therapy, exhibits bactericidal activity that rapidly diminishes the viable sputum count. Its primary effectiveness is against organisms proliferating aerobically within pulmonary cavities [184]. PZA, exhibiting unique activity under acidic conditions, is specifically tailored to exterminate organisms within caseous necrotic foci. This mechanism suggests that PZA efficacy diminishes beyond the second month of therapy [185]. RIF plays a critical role in the extermination of slowly metabolizing organisms and persisters, thereby contributing to sputum sterilization [186]. Therefore, the choice and sequence of these drugs in the treatment protocol are designed to target the full spectrum of MTB populations, addressing their diverse metabolic states and varying local environmental conditions [187]. EMB works by inhibiting the enzyme arabinosyl transferase III, responsible for the biosynthesis of arabinogalactan, therefore, it disrupts the assembly of the MTB cell wall [188].

However, therapeutic efficacy in combating MTB varies based on the bacterium location, whether it is extracellular, intracellular, or in a state of active replication. Among these, addressing the intracellular form of MTB poses the greatest challenge [189]. For anti-TB drugs to effectively target the intracellular variant of MTB, they must traverse the host cytoplasmic and phagosomal membranes, as well as the bacterial cell wall and membrane, while maintaining therapeutic concentrations [190].

In this scenario, INH stands out due to its widespread dispersion across body fluids and tissues. Its volume of distribution is roughly equivalent to 61 % body weight and its affinity for plasma proteins remains relatively minimal. The primary metabolic process for isoniazid is acetylation [191]. Its elimination half-life, including that of its metabolites, varies between 0.5 and 4 h. The primary excretion route is renal, with 75–96 % of the drug and its metabolites expelled in urine within 24 h [192]. Conversely, the challenge with RIF bioavailability is its combined administration with fixed doses of other anti-TB drugs. This can lead to the in-situ breakdown of RIF in the stomach acidic environment, a process exacerbated by INH. Recent studies described by Zivari-Moshfegh et al. [193] recognized two primary mechanisms responsible for RIF reduced bioavailability: the hastening of RIF hydrolysis and the *in vivo* RIF-quinone reaction under aerobic conditions.

The drugs used in first-line anti-TB therapy cause various side effects, with hepatotoxicity being the most common side effect [160]. Tweed et al. [194] investigated the toxicity related to the regimen of drugs in the TB treatment. The Rapid Evaluation of Moxifloxacin in TB (REMoxTB) trial was used because it is considered the most comprehensive source of safety data for standard TB therapy currently available. According to the authors, the main adverse effects related to standard TB therapy are hepatobiliary, musculoskeletal, and metabolic disorders. Recognizing that most of these effects occur in the intensive phase of treatment and mostly in female patients or patients with HIV, approximately 10 % of the total patients presented a significant side effect. These toxicities negatively affect patients and result in non-compliance and interruption of treatment, which ultimately can lead to a relapse of the infection and the MDR-TB development.

In 2022, the WHO reported that 78 % of RIF-resistant TB strains (RR-TB) were classified as MDR-TB. Responding to the escalating prevalence of MDR-TB strains globally, the WHO introduced a new 6-month treatment regimen for patients over 15 years old diagnosed with MDR or pre-XDR TB. This regimen comprises BDQ, Pretomanid, LNZ, and Moxifloxacin [7]. However, Moxifloxacin is omitted if drug susceptibility tests (DST) indicate fluoroquinolone resistance, which pertains to pre-XDR TB patients [195]. This new strategy was developed to replace the lengthier treatments for MDR-TB patients who had not been previously exposed to the drugs in the regimen. In addition, a 9-month all-oral alternative regimen, which includes BDQ, fluoroquinolones, and LNZ, was introduced to replace older treatments that had administered BDQ for extended periods [196].

Despite the evidence of efficacy regarding standardized short regimens to treat MDR-TB, there are few reports regarding the referred adverse effects. Mason et al. [197] investigated the efficacy of MDR-TB treatment in Papua New Guinea. Authors described 39 % of successful treatments and 46 % failed while 7.7 % abandoned treatment. The main cause of failures was related to the change of treatment because of the significant side effects presented, the most common being described as ototoxicity. These results are in line with those presented by Zhang et al. [198], which described how adverse effects stemming from the MDR-TB regimen led to treatment alterations in China, ultimately resulting in its failure. It was reported that 90.7 % of the patients presented at least 1 adverse effect mostly in the first 6 months, with 2 standing out as the most common; arthralgia in 67.5 % of patients and gastrointestinal disorders in 65.4 %. Other effects presented: hypothyroidism (19.7 %), dermatological disorders (17.4 %), hematological disorders (15.3 %), hepatotoxicity (11.5 %) and ototoxicity (5.9 %) while nervous system disorders such as peripheral neuropathy and psychiatric disorders appeared on average 119 days after starting treatment.

Mahata et al. [199] evaluated the adverse effects of second-line drugs to treat MDR-TB and XDR-TB. Most of the effects appeared during the first 6 months, indicating 96.10 % of the patients presented nausea, 70 % vomiting, 53.9 % digestive disorders, and 3.95 % psychiatric disorders. Pakistan is among the top 5 countries with the highest burden of MDR-TB. Javaid et al. [200] found that 70 % of patients presented psychiatric disorders after receiving treatment and 16.5 % made therapy changes. Similarly, 49.6 % of patients abandon treatment due to the adverse effects in South Korea [201].

One of the most used drugs to treat MDR-TB and XDR-TB is LNZ, which is employed in cases of resistance to RIF. In the study conducted by Cui et al. [202], the incidence of adverse effects associated with LNZ was evaluated. It was found that 27.7 % of patients experienced peripheral neuritis and 22.8 % developed hemochromatosis. An important detail is that the effects occur earlier in an interval of 45–120 days and women have a higher risk of presenting leukopenia and hemochromatosis. Among the risk factors for the use of this drug, the patient's gender, comorbidities, the duration of treatment, and the recommended doses can be considered.

Another standard regimen drug used to treat MDR-TB and XDR-TB is BDQ. Although it has proven to be clinically effective, this drug poses a high risk of generating adverse effects, some of which are considered potentially lethal [203]. Therefore, attention must be paid to the pharmacological interaction with other drugs used to treat TB, as well as the patient's clinical history. Gaida et al. [204] reported the use of BDQ in 100 % of patients without associated comorbidities successfully completed the 24-week treatment without altering or suspending the regimen due to side effects. In a study in South Africa, Padayatchi et al. [205] used a treatment based on the combination of BDQ and LNZ to treat patients co-infected with MDR-TB and HIV, at the time of the investigation all patients were on retroviral treatment. Even though 63 % successfully completed the treatment, 92 % presented adverse effects, with digestive disorders being the most common at 38.4 %, hypothyroidism 27.2 %, peripheral neuropathy 37.1 %, visual disturbances 22.5 %, anemia 25.2 %, ototoxicity 15.9 %, arthralgia 16.6 %, psychosis 14.6 %, skin disorders 11.3 %. These results demonstrate that TB therapy in patients with HIV is complicated due to drug interactions that can increase adverse effects.

Despite the implementation of the new and short regimens to treat TB, there is still a percentage of patients who abandon therapy because the treatment period is still relatively long. Furthermore, the constant and continuous presence of side effects, remain as a determining factor in the success or failure of the regimen used.

### 2.3 Economic barriers

#### 2.3.1 Financial limitations to fight against TB

The WHO's "End TB" strategy emphasizes prevention, early diagnosis, and timely treatment as key measures to lessen the global burden of TB. In 2022, the United Nations' political declaration on TB set a funding goal of $13 billion, of which only $5.8 billion was secured. For scientific research, an annual target of $2 billion was set, but merely $1 billion was invested [3].

Likewise, the global TB strategy was drastically halted during 2020–2022; since all governments gave absolute priority to stopping the advance of the Covid-19 pandemic [206]. Currently, the Pan American Health Organization asks member countries in the Americas to expand access to TB prevention, detection, and treatment services and implement access to molecular tests for the detection of drug-resistant TB. To this initiative, the WHO urges all countries to quickly implement the new BDQ, Pretomanid, LNZ, and Moxifloxacin regimen (BPaLM/BPaL) for the drug-resistant TB treatment, as it has proven to be a shorter and highly effective treatment [207].

The urgency for molecular diagnosis prior to start the TB treatment is underscored by its potential to facilitate a more precise characterization of the bacterial strain, leading to the targeted therapeutic strategy. Available molecular diagnostics encompass nucleic acid amplification tests, line probe assays, whole genome sequencing, and next-generation targeted sequencing [208]. Regrettably, these techniques are predominantly inaccessible in low-income nations due to their substantial associated costs, generating a sociological paradox as these countries concurrently exhibit the highest endemic rates and instances of drug resistance [209].

TB disproportionately affects individuals of lower socio-economic status, necessitating unfettered access to healthcare without the burden of financial constraints. It was reported in 2017 that approximately half of the global population lacks access to essential health services, with over 100 million people being thrust into extreme poverty due to exorbitant healthcare costs [210]. To quantify this predicament, the WHO devised a survey to estimate TB-related costs per patient, employing a standardized approach to gauge the impact on health systems. This investigation elucidated three primary types of expenses: direct medical costs, direct non-medical costs, and indirect costs such as loss of income. It was found that 49 % of TB patients incur these costs, amounting to over 20 % of their household's annual income [3]. Finally, the variation of these costs depending on the country and the situation of each person [211]. Dlamini et al. [212] determined that the average commercial cost for accessing MTB genome sequencing services is approximately between USD 150 and 300.

In the USA, the cost of treatment per patient can reach USD 513,000 [213]. In India, although the government offers free diagnosis and medical care for TB, it is private providers who control medical care and provide most care for TB. In 2016, the average cost per privately notified TB patient in the cities of Patna, Mumbai, and Mehsna was calculated at USD 95, 110 and 50, respectively. Veesa et al. [214] found that 34.7 % of patients sought public attention while 65 % went privately to health centers, considering that the minimum professional salary is approximately USD 78 and that around 70 % of patients made pre-treatment expenses with an average cost of USD 40.

South Africa is among the top 20 countries with the highest MDR-TB burden. It was estimated that the cost per patient of a conventional treatment of approximately 2 years is around USD 842 and the additional cost totals USD 918 [215]. In Kenya, the cost of a short TB treatment of 9 months was USD 3926.52 and for an extended regimen of 18 months was USD 8163.22, while South Africa presents a lower cost of USD 6712. The extended regimen in Russia has an approximate cost of USD 14,600, and in Philippines USD 3613 and in Peru USD 2400 [216]. The difference in medication prices, laboratory tests, and hospitalization costs generate the difference in treatment cost per patient.

In Ethiopia, even though diagnostic and treatment services are free, patients incur an average expense of USD 1378 during the diagnostic and treatment process [217]. In Ghana, the treatment cost for drug-sensitive TB was USD 429.6, while for MDR-TB it rises to USD 659 [218]. In Indonesia, the BPaL treatment per patient costs USD 7,142, in Kyrgyzstan USD 4,782, and in Nigeria USD 7,152, representing savings of 57, 78, and 68 %, respectively, compared to the total cost of conventional treatment in these countries. Thus, the gradual implementation of the BPaL regimen for MDR-TB over a period of 5 years would allow for a reduction in the national budget allocated to MDR-TB treatment by 17 % (USD 128,780) in Indonesia, 15 % (USD 700,247) in Kyrgyzstan, and 32 % (USD 1,543,047) in Nigeria [219]. Kohler et al. [220] focused on Uzbekistan, a freight cost for importing the regimen of USD 41 was estimated and the cost of the drugs was USD 1773.

This body of information illustrates the extensive economic inequality that prevails among the various nations of the globe. Such disparity can lead to insufficient treatment adherence on the part of the patient and, consequently, to its premature interruption. This situation in turn fuels the growing resistance to MTB, further exacerbating global health challenges. In light of this, the design of smart nanoscale-based therapies to tackle TB and fighting against MDR-TB comprise an excellent approach to target the diseases site, improve efficacy of diagnoses and therapies minimizing side effects.

#### 2.3.2 Nanotechnology equity

Key applications of nanotechnology, especially those aligned with the Millennium Development Goals (MDGs), disease diagnosis and detection, drug delivery systems, and health monitoring are crucial for global health. The broad application of nanomedicines across almost all medical specialties aims to enhance and extend life expectancy, particularly in low- and middle-income countries, by reducing overall healthcare costs [221].

Anticipated medical advancements from nanotechnology include the development of thermally stable drugs, ideal for use in developing countries lacking adequate storage or distribution networks. Nanosystems as new drug delivery mechanisms enable controlled and targeted drug release, improving specificity and reducing side effects. Portable diagnostic systems improve accessibility to care points with high sensitivity and specificity. However, addressing global health challenges extends beyond technological development, requiring consideration of the social and cultural contexts of countries to ensure successful implementation and acceptance of new nanomedicines. There is a global disparity in the design and application of nanosystems for disease diagnosis and treatment, accentuating health standards differences and highlighting the influence of economic resources [222].

The lack of equality in nanotechnological implementation in the medical field in low- and middle-income countries is also due to external limitations such as inadequate infrastructure, lack of equipment, unqualified personnel, and limited knowledge and confidence in this technology [223]. Thus, it is crucial to consider the entire context in which these nanosystems will be used before their development. Promoting nanotechnological development within developing countries to address their most urgent health issues through knowledge transfer is a viable solution.

The ethical responsibility to leverage advances in nanomedicine to meet major health needs, especially of vulnerable populations, rests on the global scientific community [224]. These advancements have the potential to save thousands of lives worldwide. While nanomedicines exhibit potential innovations, most studies are still on the bench or clinical phases. For further successful application, greater attention to the social and cultural contexts in which they will be used is required [225].

Point-of-care (POC) diagnostics represent a new direction in medical testing, aiming to provide patient-near care that enables rapid and accurate detection of medical conditions [226]. POC is particularly crucial in resource-limited areas, with nanotechnology playing a key role in designing effective nanodiagnostics and affordable, portable diagnostic platforms for primarily detecting infectious diseases in developing countries, where access to medical care is often limited due to economic and infrastructural constraints [227].

Nanodevices are contributing to more precise and timely diagnoses of various diseases, some of which are highly endemic and carry high mortality rates, including TB, making the development of new, fast, user-friendly, and economically viable diagnostic methods urgent [228]. In developing countries, the lack of infrastructure not only refers to biomedical and clinical equipment but also includes access to clean water, refrigeration, and electricity. Access to diagnostic tests can save lives of thousands who die from misdiagnosis and can also prevent the spread of epidemics [229].

Strategies to facilitate POC test accessibility might include developing economical, battery-operated, or solar-rechargeable devices, using local raw materials for production, and requiring minimal or no sample preparation. These new directives could significantly improve healthcare systems and disease survival rates in developing countries.

#### 2.3.3 Industrialization of nanosystems

Nanomanufacturing refers to the commercially scalable and economically sustainable production of nanoscale materials and devices, considered the fourth revolution in nanotechnology, yielding tangible results in product form. It diverges from research procedures by meeting cost, performance, and market time constraints. Scalability is a key factor slowing or even preventing the clinical translation of nanoparticles-based systems, which must be manufactured to high quality standards with batch-to-batch reproducibility and stability during long-term storage and after clinical administration [230]. Therefore, the development of efficient and cost-effective nano-scalable methods is essential. Different product specifications are considered to determine the appropriate manufacturing approach. The transition from laboratory bench to industry is underway, evidenced by nanotechnology-based products on the market. Nanodevices, particularly those that integrate artificial intelligence for real-time data acquisition and rapid processing, are commercially available, including biological and chemical nanosensors [231].

Among various nanosystems, nanoemulsions, liposomes, and solid lipid nanoparticles stand out as the most promising for swift industrial scalability, initially in developed countries and then adapted for developing countries. Nanoemulsions are transitioning from low-energy emulsification methods (used in laboratories) to high-energy methods for industrial scalability, as laboratory-scale synthesis methods significantly differ from industrial manufacturing approaches (using large-scale homogenizers), complicating their direct industrial application. Although commercially available nanoemulsions are still limited, several formulations are under advanced clinical evaluation [232].

Liposomes, extensively used in drug delivery for decades, face challenges in large-scale production with high reproducibility. However, new methods for simple reproducibility and industrial scalability have been developed, as demonstrated by the first report indicating that preserving liposomes in dry film state for subsequent *in situ* hydration exhibits excellent yields, emerging as a promising large-scale production technology [233]. Most liposome fabrication methods require additional steps to ensure their homogeneity, so scalable methods must avoid size variability in production. Formulation methods leveraging fluid control and lipid mixing facilitate one-step liposome preparation, reducing variability and enhancing stability for reproducibility [234]. This discovery also suggests that microfluidic equipment can be designed and scaled for high-throughput production in developing countries. Another challenge for liposomal drugs is the additional processing step required to remove unencapsulated drugs, which are highly costly and low in scalability. Economical and rapid solutions for scalable drug production have been developed, as shown by using egg phospholipids to separate unencapsulated drugs [235].

Regarding polymeric nanoparticles, a novel flow chemistry reactor has demonstrated the synthesis of various nanodrugs, including clindamycin-loaded chitosan nanoparticles, with significantly reduced particle size distribution and overall smaller size. The high productivity of this technique suggests its potential for industrial-scale nanoparticle manufacturing [236].

Solid lipid nanoparticles (SLNs) offer an alternative carrier system to liposomes, polymeric nanoparticles, and inorganic carriers. SLNs have gained increasing attention in recent years for delivering drugs, nucleic acids, proteins, peptides, nutraceuticals, and cosmetics. Their economic production, simple preparation, and desirable physicochemical stability for industrial scalability make SLNs promising nanodrugs for large-scale manufacturing. Several SLN-based products are in clinical trials, with a high likelihood of rapid market presence increase [237].

The integration of nanotechnology in medical and industrial applications marks the beginning of a transformative era, especially in healthcare and manufacturing. The alignment of nanomedicines with the MDGs highlights its potential to revolutionize disease diagnosis, drug delivery, and health monitoring, particularly in low- and middle-income countries. The ethical imperative for the global scientific community is to ensure that advances in nanotechnology are accessible to all, thereby addressing urgent health challenges and contributing to the industrial revolution.

## 3 Nanoscale drug delivery systems

**Fig. 4:**
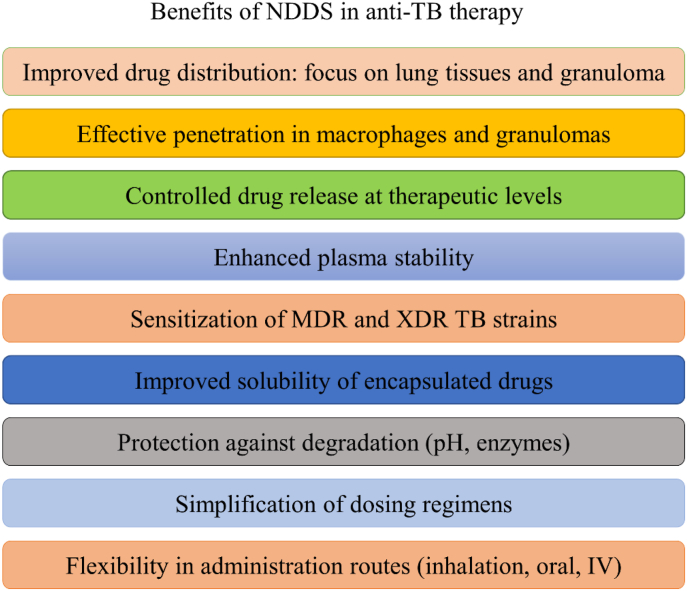
The potential of NDDS for anti-TB therapy.

In recent decades, nanoscale drug delivery systems (NDDS) have emerged as a promising therapeutic in the fight against TB. NDDS present significant advantages over conventional formulations, including the potential to improve the bioavailability of anti-TB drugs (Table 2). The benefits provided by NDDS extend beyond merely enhancing bioavailability (Fig. 4). In *in vivo* trials, they have proven effective increasing of targeted drug delivery, reducing systemic toxicity, and providing sustained drug release in the plasma (Table 3). This breakthrough revolutionizes the landscape of pharmacological therapies by providing controlled drug release, thereby improving its therapeutic effectiveness [238].

**Table 2:** Comparative analysis of conventional versus NDDS therapies.

Aspect	Conventional therapy	NDDS therapy
Drug Distribution	Less precise, may affect non-target areas	Highly precise, improves targeting of affected tissues
Targeted Delivery	Limited, mainly systemic distribution	Enhanced, allows direct targeting of pathogens or disease sites
Drug Release	Rapid, may require frequent dosing	Controlled and sustained, reducing the need for frequent dosing
Side Effects	Higher, due to systemic exposure	Reduced, targeted delivery minimizes exposure to healthy tissues
Adherence to Treatment	Lower due to complex regimens and side effects	Improved through simplification of dosing and reduction of side effects
Cost-effectiveness	Varies, but generally lower initial costs with potentially higher indirect costs	Potentially higher initially but can lead to lower healthcare costs due to increased efficacy and reduced dosing frequency
Flexibility in Administration	Limited, mainly oral and injectable	Greater, includes inhalation, oral, and injectable
Stability and Solubility	Variable, many drugs have solubility and stability issues	Improved, nanoencapsulation may enhance solubility and protect the drug from degradation

**Table 3:** Recent developments in nanosystems targeting MTB with *in vivo* applications. Data Source: SciFinder, utilizing the search terms “*Mycobacterium tuberculosis*”, further refined with “nanoparticles” and “*in vivo*”. Review articles, articles published before 2018, and those containing the keyword “Vaccine”, as well as preprint articles, were excluded from the research.^a^

Nanosystems	Size (nm)	*In vitro* effect	Dose and animal model	*In vivo* effect	Reference
ZnO Nanoparticles (ZnNPs)	N/A	MIC between 0.5 and 2 mg/L. Changes in autophagy and ferroptosis markers in BCG-infected macrophages	Female C57BL/6J mice, 500 mg/(kg⋅day)	Worsening of lung lesions and increased inflammatory cytokines. Ferroptosis blocker reduced inflammation	[244]
Clofazimine Nanocrystals (CLF-NCRs)	∼600	MIC of ∼0.62 μg/mL	BALB/c mice, oral administration 25 mg/kg	Significant reduction in CFU and improvement in lung histology after 30 days with CLF-NCRs	[245]
Amorphous Nanoparticles (BDQ/BTZ)	60 ± 13 (BDQ), 62 ± 44 (BTZ)	Ability to penetrate biological barriers and reach intracellular locations of mycobacteria	C3HeB/FeJ mice, Intranasal administration 6.56/6.72 mg/kg	High concentrations of BDQ/BTZ in lung granulomas with minimal presence in spleen or liver	[246]
CS-PLGA -β-glucan Nanoparticles (CS-PLGA@GL)	∼217	Increased gene expression for specific cytokines at certain β-glucan concentrations. ROS production and specific protein secretion also increased. Significant reduction in intracellular MTB accumulation in macrophages	BALB/C mice, via oropharyngeal aspiration 31.25 μg/g body weight	Both CS-PLGA@GL and free β-glucan increased specific cytokine levels in male CD1 mice. However, free β-glucan only matched the nanoparticle effect at higher concentration	[247]
Cellulase and Levofloxacin-loaded Composite Nanoparticle (CL@LEV-NPs)	196.2 ± 2.89	Enhanced anti-biofilm effects when combined with ultrasound due to ROS generation	BALB/c mice, 5 mg/kg	CL@LEV-NPs facilitated the removal of biofilms from subcutaneous implants in BALB/c mice post-BCG infection and reduced local inflammation	[248]
PeptoMicelles	98–100	Micelle stability and low potency against *Mycobacterium marinum*	Intravenous administration 180 mg/kg	Comparable efficacy to free drug in C3HeB/FeJ mouse model. Safety confirmed in zebrafish larva model	[249]
RIF-loaded iron oxide nanoparticles with polyacrylic Acid polyethylene glycol coating and mannose functionalization (Rif@IONPs-PAA-PEG-MAN)	20–22	Rif@IONPs-PAA-PEG-MAN showed high biocompatibility with minimal cytotoxicity and were preferentially internalized by MTB-infected macrophages, involving mannose receptor interaction, endocytosis, micropinocytosis, and phagocytosis pathways. The majority of intracellular IONPs localized to acidic lysosomes, facilitating the release of RIF under acidic pH conditions. This enhanced uptake and RIF accumulation in macrophages led to consistently elevated intracellular RIF levels compared to free RIF-treated cells	Female BALB/c mice, aged 8 weeks, were infected. RIF@IONPs-PAA-PEG-MAN group was treated with 200 μL, 5 mg/kg for oral administration	Direct exposure to high RIF levels and potential excess iron release from RIF@IONPs-PAA-PEG-MAN diminishes intracellular MTB viability. This nanosystem augments innate immune elimination of intracellular MTB by promoting M1 antimicrobial polarization in infected macrophages, boosting proinflammatory/anti-TB cytokine production like TNF-α	[250]
Polymeric Micelles	84–149	MIC90 between 0.016 and 0.5 for five water-insoluble nitronaphthofuran derivatives solubilized in polymeric micelles	Zebrafish model: 5 mg/kg	Encapsulated compounds increased embryo survival by 66–70 % eight days post-infection with a high dose of 50 mg/kg	[251]
LNZ-loaded mannosylated gelatin nanoparticles (MAN-GNPs@LNZ)	197–298	Enhanced LNZ concentration in alveolar macrophages due to mannose receptors	Wistar rats, 10 mg/kg of LNZ in 5 % w/v gelatin solution	MAN-GNPs@LNZ reduced dose, frequency, adverse effects, and prolonged presence in lungs	[252]
Nanopolymeric Coordination Systems (SNPC)	∼500	INH/diclofenac (INHDIC) in SNPC inhibited MTB growth in host cells (RAW 264.7) and showed potential against infection. INHDIC also reduced migration in the A549 lung cancer cell line	BALB/c mice, INHDIC in PBS suspension with variable doses (5, 25, 50, 100 mg/kg)	SNPC-INHDIC was safe up to 100 mg/kg in BALB/c mice. It demonstrated sustained drug release and potential as a multi-drug delivery system	[253]
Mannosylated Preactivated Chitosan Nanoparticles (MAN-CS-NPs)	307.6	MAN-CS-NPs enhanced the oral bioavailability of RIF by 16-fold	Swiss albino mice, Oral dose of 12 mg/kg	Improved oral bioavailability of RIF with NPs in rabbits. Normalized transaminases and increased glutathione levels	[254]
Mannose-modified macrophage-targeting SLN MAN-SLN@INH	236	Inhalable and pH-sensitive MAN-SLN@INH improved intracellular antibiotic efficacy against MTB	Wistar rats, Inhaled administration 3 mg/rat	MAN-SLN@INH showed greater antibiotic efficacy in Wistar rats than the INH solution. Potential against latent TB highlighted	[255]
Polycationic Phosphorus Dendrimers (PPD)	N/A	21 PPDs synthesized; 2 showed promise for MDR TB with MICs between 3.12 and 6.25	BALB/c mice, oral dose of 50 mg/kg or 33 mg/kg in water	Significant reduction in pulmonary bacterial load in BALB/c infected mice. Superior efficacy compared to EMB and RIF	[256]
MCC7433-loaded mesoporous silica nanoparticles	∼100-200	The nanocarrier demonstrated the best potential for enhancing the aqueous solubility of MCC7433 (novel nitroimidazopyrazinone analog), showing a MIC 90 of ≤0.25 μg/mL	MSN orally administered to CD-1 male mice at a dose of 20 mg/kg using a gavage procedure	MCC7433-loaded nanocarriers improve systemic exposure in mice, yielding a 1.3-fold higher Cmax compared to free MCC7433. This enhancement indicates their potential as nanocarriers for poorly soluble anti-TB drugs.	[257]
RIF-loaded Liposomes	137	N/A	In this study, the guinea pigs were exposed to an inhalation dose of 2.83 mg/kg	Significant reduction in inflammation levels, inferred from the organ weights, and a decreased bacterial load in the lung regions of the guinea pigs, suggesting that the nanocarrier enhances granulomatous penetration	[258]
Hydrophilic Streptomycin Sulfate SLNs (STRS-SLN)	218.1	Enhanced intracellular absorption in THP-1, LoVo, and DLD-1 cells. 3-fold MIC reduction against MTB H37RV compared to free STRS	Wistar rats, oral administration 90 mg/kg	STRS-SLN plasma concentration surpassed the STRS group. Oral administration improved adherence and reduced costs	[259]
Alginate-coated Silver Nanoparticles (ALG-AgNPs)	70	TB-MDR strains had specific MICs. Astonishingly, sensitive strains had MICs between 4 and 41 μg/mL	BALB/c mice: various doses orally or intravenous injection	ALG-AgNPs demonstrated *in vivo* safety and potent anti-mycobacterial effects in zebrafish and BALB/c mice models	[260]
Benzothiazinone-loaded human serum albumin nanocarriers (dBTZ HSA-NP)	60–130	dBTZ HSA-NP achieved a MIC of 0.3 μM in intramacrophage MTB	C3HeB/FeJ mice, Intranasal administration of specific doses daily for 10 days	Pulmonary administration of dBTZ achieved significant reductions in bacterial load (<2 × 10^6^ CFU) compared to the untreated control group, with lower doses and shorter therapeutic duration	[261]
Culture Filtrate Proteins - Chitosan Nanoparticles (CS-CPF)	∼250–∼300	N/A	BALB/c mice treated with specific doses via intraperitoneal for 21 days	CS-CPF provided protection over 60 days compared to free CPF. Controls showed a 95 % higher MTB growth compared to treated mice	[262]
Levofloxacin-loaded PLGA nanoparticles (LN-NP)	146	MIC for levofloxacin was 1.0 μg/mL and for LN-NP was 0.8 μg/mL	BALB/c mice, dose not reported	The AUC 0-∞ of LN-NP in the lungs of BALB/c mice was 380.2 μg/gh for the test group and 69.68 μg/mlh for the control group, and drug release was maintained up to 120 h	[263]
Rifampicin-loaded biodegradable polyester n anoparticles (BP-NP-RIF)	∼34	N/A	BALB/c mice, 10 mg/kg	BP-NP-RIF showed excellent tolerability and significantly greater efficacy compared to equivalent doses of free RIF	[264]
Mannosylated Gelatin Nanoparticles of Licorice (MAN-GE-NP@LE)	300	Mannosylated formulation enhanced cellular absorption. Significant CFU reduction after applying specific concentrations of MAN-GE-NP@LE in U937-infected macrophages	BALB/c mice, specific doses over 8 weeks	The number of tubercles was significantly lower in the lungs of animals in the standard drug treatment group and the group treated with MnGNP compared to the untreated control group and animals treated with blank MAN-GE-NP@LE	[265]
Genipin-crosslinked CS/INH/RIF Nanogel (GEN-CS/INH/RIF NGPs)	60–130	GEN-CS/INH/RMP NGPs showed a pronounced additive antibacterial effect against specific strains	Wistar rats, Intrapulmonary administration for 7 days at specific doses	Intrapulmonary application of GEN-CS/INH/RIF NGPs resulted in therapeutic drug concentrations in the lungs and reduced presence in other organs. Demonstrated lung-targeting properties and reduced toxicity	[266]
Spontaneous Extracellular Vesicles (S-EV)	130	Reduction in mycobacterial load and decreased MCP-1 and TNF-α production in specific macrophages	BALB/c mice, specific doses	Bacterial load reduced by one-third after 60 days of treatment	[267]
NZX Peptide-loaded mesoporous silica nanoparticles (NZX-MSN)	200	Achieved 80–100 % internalization of MSN in macrophages and THP1 cells depending on concentration	BALB/c mice, intratracheal administration after 19 days of infection, dose not reported	An 84 % decrease in CFU counts was observed in the NZX-treated group, 88 % in the MSN-NZX treated group, and a significant 90 % reduction in the rifampicin-treated group.	[268]

a Abbreviations: not applicable (N/A); 1,3-benzotiazin-4-ona (BTZ); chitosan (CS); lactic-co-glycolic acid (PLGA); β-glucan (GL); Area Under the Curve (AUC); Colony Forming Units (CFU); Polyethylene Glycol (PEG); Polyacrylic Acid (PAA).

**Fig. 5:**
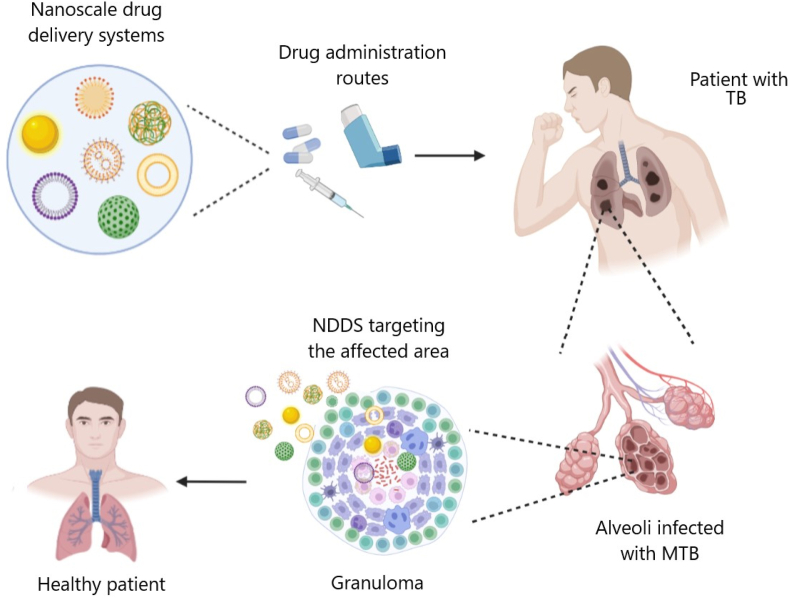
The NDDS employ various drug administration routes to specifically treat areas affected by TB. Created in biorender.com.

Advances in nanotechnology have opened new avenues for drug administration. Inhalation aerosols represent a significant advance in this field, allowing selective biodistribution to the lung, one of the organs most affected by TB [239]. NDDS are particularly useful in the treatment of latent TB infection as they can facilitate penetration into granuloma tissues (Fig. 5). This property is essential to address the resistance to penetration of conventional drugs often presented by these tissues [9].

Various types of NDDS-loaded anti-TB drugs, including nanoemulsions [240], lipid nanoparticles [241], metal-based nanoparticles [242], polymeric nanoparticles [240], and mesoporous silica nanoparticles [243]. These nanosystems have presented set of advantages and challenges towards the optimization of new approaches for TB treatment.

### 3.1 Nanoemulsions

Nanoemulsions (NE) are nanoscale liquid-liquid dispersions that exhibit the ability to increase drug solubility, stability, and bioavailability, as well as enhance the activity of existing drugs [269,270]. These nanosystems are composed of water, oil, an emulsifying agent, and a co-emulsifier, their production can be carried out through high or low energy procedures [271]. As drug delivery vehicles, NE play a crucial role in optimizing therapeutic efficacy while minimizing adverse reactions and potential toxicities associated with the provided drugs [272]. In the context of oral administration, these NE enhance the solubility of lipophilic drugs, promoting prolonged residence in the GIT and promoting more effective lymphatic absorption [273]. In this way, NE provide an approach to avoid the first-pass metabolism of the drug. However, it is essential to take into account certain peculiarities when manipulating NE, as they represent thermodynamically unstable systems that tend to separate rapidly into two markedly different phases, unless molecules with surface activity, known as emulsifiers, are included in the mix to ensure the nanosystems stabilization [274].

**Fig. 6:**
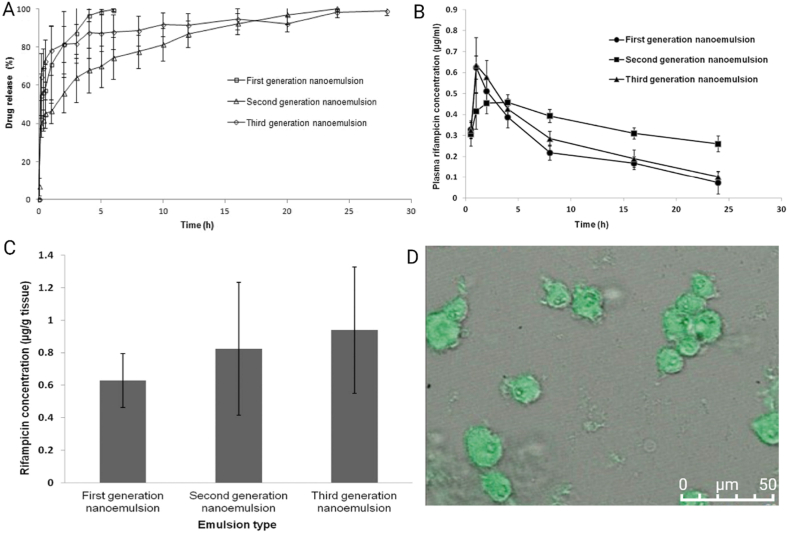
A) Profile of the cumulative percentage of RIF release B) Mean concentration-time curves for RIF in plasma, C) Concentrations of RIF in lung tissue homogenates, D) Confocal laser scanning microscopy (CLSM) image demonstrating nanoemulsion endocytosis [276]. Reprint published from open access article by Taylor & Francis Copyright 2017.

Given that MTB survives within phagocytes, it is strategic to target drugs specifically to alveolar macrophages. Chitosan, composed of glucosamine and n-acetylglucosamine units, serves as an effective ligand for mannose receptors on macrophages harboring mycobacteria due to its n-acetylglucosamine residue [275]. In this context, Shah et al. [276] prepared NE using the spontaneous emulsification method. A first-generation rifampicin-loaded oil-in-water (o/w) NE, utilizing oleic acid as the oil phase, ethanol as the drug co-solubilizer, Tween 80 as the surfactant, and normal saline as the aqueous phase. Subsequent generations of NE, namely the second and third, were developed by modifying the oil droplets of the initial formulation. These enhancements involved coating the droplets with oligochitosan or an oligochitosan-folate conjugate at various concentrations (0.1, 0.25 and 0.5 %), thereby equipping the NE with targeting capabilities aimed at alveolar macrophages. Characterization included evaluations of viscosity, pH, surface tension, hydrodynamic average particle size (from 40 to 60 nm), zeta potential (from −2.5 to + 4.18 mV) and initial *in vitro* release followed by sustained release (Fig. 6A), which could eradicate non-phagocytosed bacteria in the lungs, while the sustained drug release phase could be beneficial in eradicating phagocytosed bacteria in alveolar macrophages. The formulations exhibited high aerosolization performance (90 %) with a high drug content (95 % aerosol output), low RIF concentration in the bloodstream up to 24 h (Fig. 6B), with up to 85 % of the nanosystems reaching pulmonary deposition (Fig. 6C). Confocal laser scanning microscopy (CLSM) confirms that the formulations can be internalized in macrophages without compromising cell viability (Fig. 6D), which is crucial for TB treatment. In fact, the RIF-loaded chitosan-folate modified NE exhibited greater internalization due to the dual receptor targeting (mannose and folate receptors, overexpressed in macrophages). Together, these findings represent an important tool for local drug administration to treat pulmonary TB.

Similarly, Bazán-Henostroza et al. [277] also proposed NE for the local treatment of ocular TB, using a cationic RIF-loaded NE (prepared by high-pressure homogenization with oleic acid, poloxamer 188 and polysorbate 80). Ocular TB can cause permanent vision loss and the current therapy is oral RIF administration, which causes serious side effects. In ocular TB, another key factor is the presence of the blood-retinal barrier, which limits the diffusion and bioavailability of drugs in the eyes. The cationic NE exhibited an average hydrodynamic size of around 150 nm, obtained by modifying the surface of the conventional oleic acid NE with chitosan and polymyxin B, significantly modifying the net charge of the nanosystem. Chitosan- or polymyxin-modified formulations (+51.3 and + 5.5 mV, respectively) showed strong interaction with mucin (negatively charged) through hydrogen bonding and hydrophobic association forces, compared to conventional NE. The results corroborate the less frequent instillation of the product due to the longer retention time. The formulations maintained their anti-TB activity against MTB after their preparation and modification. This strategy presents a suitable formulation for ocular TB treatment and for opportunistic bacterial co-infection, allowing the simultaneously RIF delivery to a hydrophilic antibiotic like polymyxin B.

Hussain et al. [278] proposed to design another cationic NE, but in this case as a gel for transdermal RIF administration, as an alternative for the treatment of both cutaneous and systemic TB. The NE, prepared with capmul PG8 (lipid), labrasol (surfactant), and acconon MC8/transcutol (co-surfactan) by a slow and spontaneous emulsification, showed suitable physicochemical properties such as small droplet size (<100 nm), great encapsulation efficiency (EE = 60 %) and positive charge (+32.81 mV). The nanogel formulation (NE + 1 % carbopol gel) presented a high skin permeation and drug deposition compared to the solution or conventional NE. *In vitro* studies also demonstrated that the NE is hemocompatible. Both the conventional NE and the nanogel showed sustained release over time. An *in vivo* study with Sprague-Dawley rats demonstrated that the maximum plasma concentration after skin nanogel administration was almost 5 times higher compared to the drug suspension administered orally, indicating that the nanogel is a promising vehicle to increase the bioavailability of RIF by transdermal use.

### 3.2 Liposomes

Liposomes (LPs) were discovered by Alec Bangham in 1965, quickly taking a leading role in drug delivery [279]. Their spheroidal structure, which comes from the self-assembly of amphipathic molecules into a bilayer, provides exceptional ability to load and deliver a variety of pharmaceutical compounds, which can be both hydrophilic and hydrophobic molecules [280].

LPs have high biocompatibility and biodegradability, as they are composed of lipids and fatty acids that enable them to mimic natural cellular membranes and promote effective cellular uptake [281]. The liposomal membrane, formed by one or more lipid bilayers arranged around an aqueous core, gives liposomes the ability to carry molecules of distinct solubilities. The fluid lipid surfaces facilitate the functionalization of specific ligands, and the adjustability of these nanoplatforms allows them to easily alter their shape [282].

An important advantage of LPs is the ability to transport both hydrophilic and hydrophobic drugs, such as INH and RIF. Additionally, LPs have suitable technological properties, are biocompatible, and have wide versatility to be included in other pharmaceutical forms, such as gels (transdermal administration) or aerosols (dry powder) for pulmonary instillation [278,283]. In addition, liposomes significantly enhance stability and demonstrate an unparalleled ability to traverse challenging biological barriers such as the intestinal membrane, macrophages, and the blood-brain barrier [284].

**Fig. 7:**
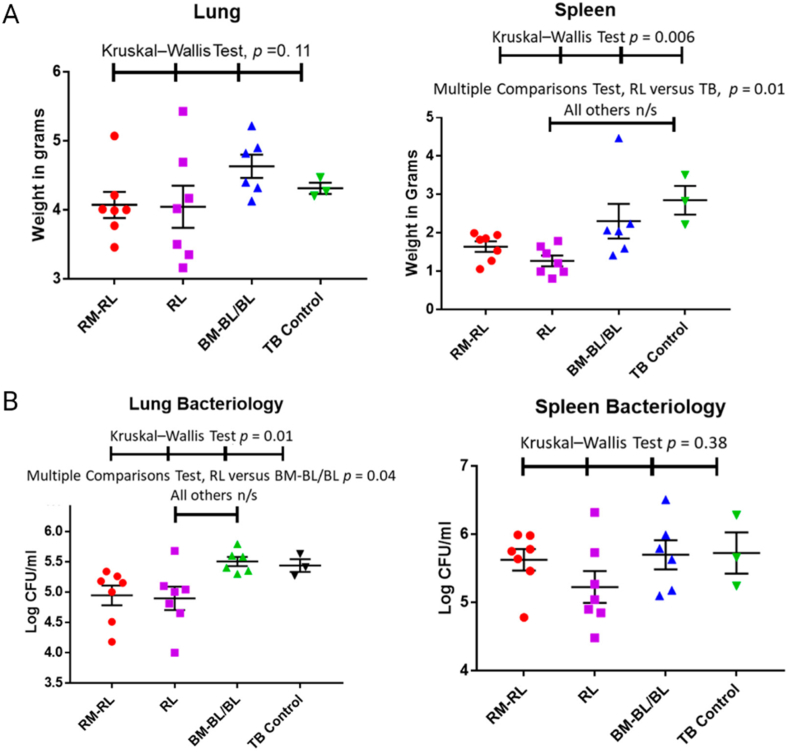
A) Wet organ weights of animals infected with tb based on the treatment, B) Number of viable bacteria in the lung and spleen tissues of the animals at necropsy after 10 days of treatment. RIF-loaded microparticles (RM); RIF-loaded liposomes (RL); RM-RL association; control for microparticles and liposomes (BL-BM) [285]. Reprint published from open access article by MDPI Copyright 2021.

García-Contreras et al. [285] developed two systems: RIF-loaded liposomes (comprising L-α-phosphatidylcholine and cholesterol) and RIF-loaded PLGA microparticles (by the emulsion/solvent evaporation method). They individually assessed the penetration capabilities of both nanosystems into granulomas. The liposomal formulation exhibited a spherical shape and a smooth surface (Fig. 7A), with an average hydrodynamic diameter of 137 nm. This formulation was applied to guinea pigs infected with TB via inhalation at a dose of 2.83 mg/kg. The observations meticulously unveiled a discernible attenuation in inflammation levels, inferred from organ weights, and a diminished bacterial burden in the pulmonary regions of the treated cohorts. However, it is noteworthy that in the spleen, no significant disparities were detected (Fig. 7B). These insights collectively suggest an enhancement in RIF's granulomatous penetration attributable to the liposomal delivery, underscoring its potential in targeted pulmonary applications. However, the authors highlighted a limitation in using PLGA microparticles in the nebulization process due to their larger size and propensity for aggregation. This emphasizes the significance of particle size in achieving effective granuloma penetration and, consequently, successful TB treatment.

**Fig. 8:**
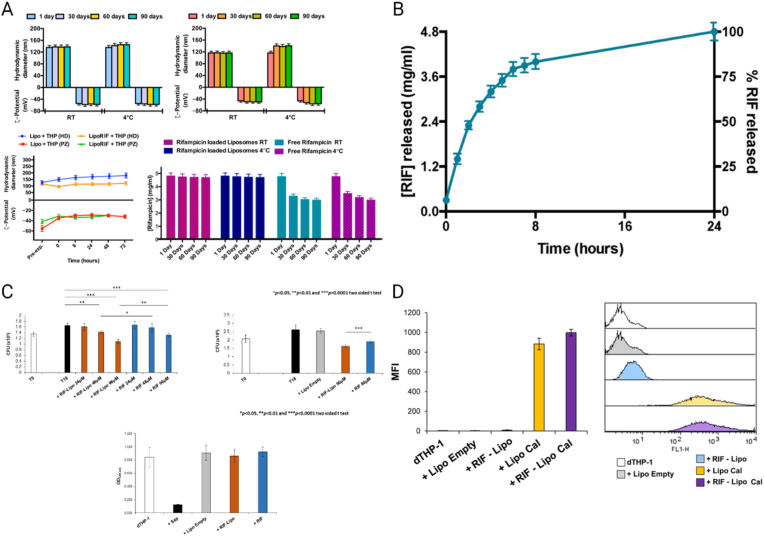
A) Physicochemical stability over time under room temperature conditions and at 4 °C, and how these parameters can affect the hydrodynamic diameter and zeta potential. The influence of the THP culture medium on these parameters was also evaluated by varying incubation intervals. B) Release pattern of RIF over 24 h, C) Enhanced intracellular killing of mycobacteria by RIF-loaded liposomes without any cytotoxic effects and D) Analysis of liposome internalization within macrophages [286]. Reprint published from open access article by MDPI Copyright 2021.

Rinaldi et al. [286] developed RIF-loaded calcein LP by thin-layer evaporation technique (using Hydrogenated phosphatidylcholine and 1,2-Dipalmitoyl-sn-glycero-3-phosphorylglycerol) for pulmonary administration. The LP formulation exhibited average size up to 130 nm, and a strong negative zeta potential (−55.4 mV) with good physicochemical stability for 90 days, and desirable drug content (∼100 %) at different temperatures and in THP medium (Fig. 8A). The LP showed controlled drug release over 24 h (Fig. 8B). An *in vitro* model of macrophage infection was performed using a human pro-monocytic leukemia cell line THP-1 infected with *Mycobacterium abscessus*; the results indicate a greater reduction of intracellular mycobacterial viability for the liposomal formulation, compared to free RIF, with no toxic effect on the macrophages (Fig. 8C). Cellular absorption analysis showed that LPs could be efficiently internalized by the macrophage. For these studies, THP-1 cells (5 × 10^5^ cells/mL) were stimulated for 18 h with either pristine liposomes (Lipo-Empty) or RIF-loaded liposomes (RIF-Lipo), which either contained calcein (Cal) or did not. Subsequently, the cells were collected and the uptake of liposomes was analyzed by flow cytometry. The results were expressed as mean fluorescence intensity (MFI) (Fig. 8D). These data revealed the potential of the nanoplatform to increase the intracellular RIF activity, with considerable benefit in the treatment of patients with deep pulmonary infection.

### 3.3 Solid lipid nanoparticles and nanostructured lipid carriers

In 1990, Müller, Gasco and S. Lucks developed independently SLN with the main goal of replacing the traditionally used organic solvents in the synthesis of polymeric nanoparticles [287]. Comprising solid lipids and surfactants, SLNs exhibit superior stability compared to liposomes. They can solidify at body temperature and release drugs in a controlled manner, providing protection against drug degradation. Additionally, they evade the adverse effects associated with the use of organic solvents and offer improved stability and tolerability compared to polymeric nanosystems [288]. However, they have limited drug loading capacity due to their strict solid lipid composition and internal crystalline structure reconfiguration [289,290].

In response to the limitations of SLNs, NLC were developed and represent a sophisticated second generation in the realm of lipid-based drug delivery systems [291]. NLCs are characterized by their multifaceted composition, which incorporates both solid and liquid-state lipids in addition to emulsifiers, all with biodegradable and biocompatible properties [292]. This unique blend enhances the drug transport capacity and prevents its premature release. Adding liquid lipids to NLC infrastructure causes structural anomalies in solid lipids, resulting in a less ordered crystalline arrangement that avoids early drug expulsion, ensuring high drug loading and thus optimizing therapeutic delivery [293].

Most studies on lipid NDDS for anti-TB drugs refer to SLN and NLC since they became very popular over the last two decades and have outstanding biopharmaceutical properties for drug administration [294]. Several authors reported the development, optimization, and characterization of SLN containing INH and RIF, emphasizing suitable physicochemical properties for anti-TB drug administration [295,296]. These properties include small size, biocompatibility, sustained drug administration, pH-dependent drug release, good shelf-life, and acid stability, enhanced pharmacokinetic properties, greater biodistribution, and pulmonary retention achieved through surface modification (*e.g*., with lactoferrin), as well as increased cellular uptake in macrophages (with chitosan and mannose-like surfactant modification) [297,298]. This is achieved without causing toxicity to macrophage cells and by reducing the inherent toxicity of free drugs in non-target organs, such as the liver and spleen. Additionally, authors have making efforts towards the design of inhalable aerosols-based formulations for pulmonary drug administration [299]. Together, these characteristics confirm the role of SLN and NLC as safe and effective nanotools, capable of improving local administration and the safety of anti-TB drugs with limited side effects.

**Fig. 9:**
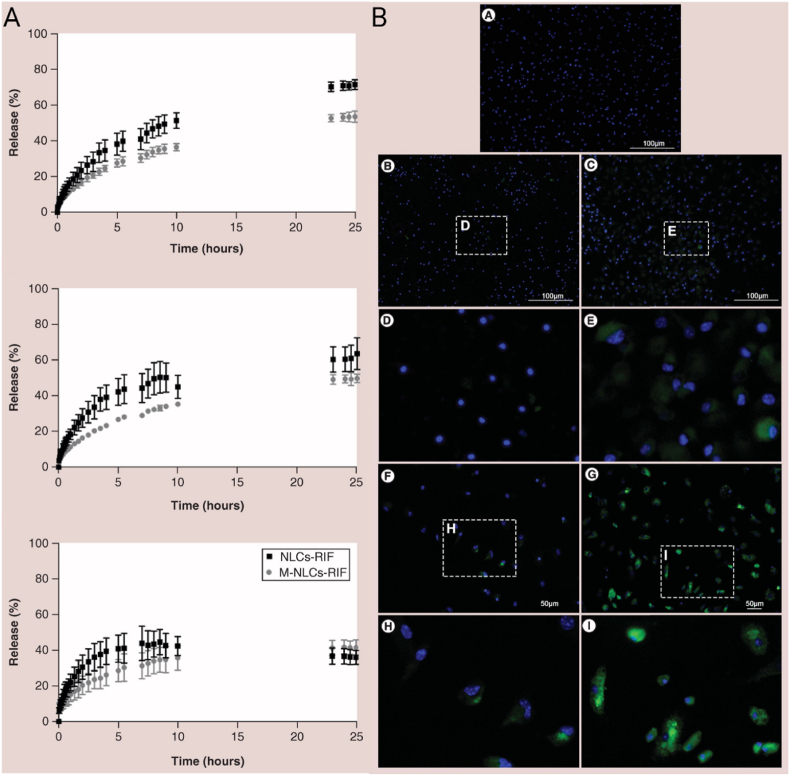
A) The release profiles of RIF-loaded formulations were meticulously analyzed *in vitro*, considering the progression of time and the simulation of different environments including the lung fluid, the phagosome, and the phagolysosome. B) The mannosylated NDDS conjugated with fluorescein isothiocyanate showcased enhanced efficiency in terms of internalization within primary macrophages. Reprinted/adapted with permission from Ref. [300]. Copyright 2017, Future Medicine Ltd.

In this context, Vieira et al. [300] synthesized mannosylated RIF-loaded NLC (RIF-NLC/M) combining the techniques of high shear homogenization and ultrasonication (using solid lipid 58 %, liquid lipid 25 %, surfactant 16 %, and 1 % RIF) for the TB treatment. The RIF-NLC/M presented a narrow size distribution (∼315 nm), a strong positive zeta potential (+36 mV) with high drug encapsulation (>90 %). The release of RIF-loaded nanosystems was studied over time, considering the pH levels found in pulmonary liquid (pH 7.4), phagosomes (pH 6.2), and phagolysosomes (pH 5.0) (Fig. 9A). M-NLC-RIF provided 14 times increase in cell uptake in bone marrow-derived macrophages (BMDM) compared with NLC-RIF (Fig. 9B). An *in vitro* model of intracellular macrophage infection using BMDM and *Mycobacterium avium* was performed, which showed that the M-NLC-RIF has superior cytotoxic properties compared to conventional NLC-RIF, corroborating the cell uptake data. The method developed by the authors allowed obtaining a tool capable of improving intracellular RIF release in macrophages, an important strategy for eliminating intracellular MTB.

Another study evidenced the use of NLC formed with stearic acid and oleic acid and emulsified with Tween 80, RIF and tuftsin-modified peptide (pRIF-NLC). This peptide can be used as a target as it tends to recognize overexpressed macrophage receptors, involved in site-specific macrophage infection, such as TB. pRIF-NLC showed a narrow size distribution (∼285 nm) and a negative zeta potential (-22 mV). The stability of the nanosystems were evaluated and the samples maintain their physicochemical properties for 60 days. *In vitro* release confirmed sustained release over 72 h. This pRIF-NLC formulation showed a 2-fold increase in cell uptake in murine macrophages: J774 A.1 cell line, suggesting that RIF-NLC has desirable technological properties for improving intracellular drug administration in macrophages. pRIF-NLC formulation showed significant reduction of the cytotoxic effects of RIF after encapsulation and growth inhibition in *in vitro* cultures of the MTB strain H37RV. The adaptation using tuftsina-loaded NLCs led to a diminished RIF cytotoxicity, while augmenting its specificity and uptake by alveolar macrophages. This constitutes a pivotal advancement towards safer and more precise therapies, forging new pathways to tackle TB and infectious diseases [301].

Ma et al. [302] developed an inhalable mannose-modified macrophage-targeting solid lipid nanoparticles (MAN-IC-SLN) to achieve intracellular drug administration in MTB infected macrophages. The MAN-IC-SLN based on tearyl amine, palmityl palmitate, poloxamer188 and mannose were prepared and loaded with INH. The *in vitro* release was pH-dependent showing 82 and 58 % of release in pH 5.5 and 7.4, respectively. MAN-IC-SLN showed twice the cell uptake in murine macrophage cells (Raw 264.7). MAN-IC-SLN did not exhibit a cytotoxic effect in A549 (human basal alveolar epithelial cells of adenocarcinoma) and Raw 264.7 macrophage cells. *In vitro* experiments, MAN-IC-SLN showed a 4-fold higher antibacterial effect against *Mycobacterium smegmatis*. In addition, the MAN-IC-SLN exhibited greater antibacterial effect on intracellular bacteria than on extracellular bacteria, possibly associated with greater cell uptake, while it has a low antibacterial effect on extracellular bacteria, probably associated with pH-dependent INH release. MAN-IC-SLNs were successfully nebulized into a fine particles fraction with high inhalation capacity, showing desirable lung deposition. The *in vivo* antibiotic effects were evaluated using a model of *Wistar* rats, infected with *Mycobacterium smegmatis*, which showed that the MAN-IC-SLN had the strongest antibiotic properties compared to free INH, possibly due to greater lung deposition. This result in a new possibility to increase the intracellular deposition of drugs in TB-infected macrophages for the latent MTB treatment.

**Fig. 10:**
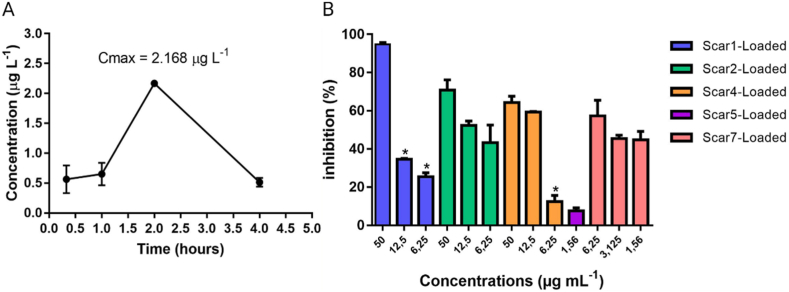
A) Temporal plasma concentration profile of Scar2-loaded NLS following a single oral administration via gavage at a dose of 300 mg/kg body weight, B) Intramacrophage activity of Scar-loaded nanosystems [303]. Reprint published from open access article by Frontiers Media S.A. Copyright 2018.

Likewise, Da Silva et al. [303] investigated the antimycobacterial efficacy of Ru(II) heteroleptic compounds, known as SCARs. They found that these compounds maintain their efficacy when encapsulated in SLNs (labeled SCAR-SLN, composed of 10 % cholesterol, 10 % phosphatidylcholine, sodium oleate, and Eumulgin HRE 40, with 80 % of the formulation being phosphate buffer). The SLNs were prepared using the sonication method. The hydrodynamic average particles size ranged from 171.6 to 213.0 nm, and the zeta potential from +0.305 to +0.581 mV. The complex encapsulation resulted in low cytotoxicity, which is attributed to the complex controlled release and the presence of cholesterol, improving the interaction with the bacterial membrane. SCAR-SLN were found to be effective against various MDR-TB strains, with no evidence of resistance to these compounds. In particular, the SCAR4-SLN stood out by maintaining a stable MIC90 among the analyzed strains, regardless the solubilization medium. Oral bioavailability assays indicate that SCAR2 reached a concentration of 2.7 μg/mL in plasma 2 h after its administration (Fig. 10A). In addition, the authors highlighted the intramacrophage efficacy of SCARs, demonstrating their capability to suppress MTB proliferation inside macrophages and achieving up to 90 % MTB inhibition at concentrations as low as 1.56 μg/mL (Fig. 10B). These results suggest that the nanosystems can play an important role in the fight against MDR-TB, both at the extracellular and intracellular levels.

### 3.4 Metallic nanoparticles

Recent strides in the study of metallic nanoparticles (MNPs) have impacted the biomedicine landscape, attributable to their exceptional adaptability in diagnostics and therapeutic applications [304]. MNPs exhibit an array of distinguished properties: an enhanced electromagnetic field at their surface, a comprehensive suite of optical characteristics, efficient synthesis routes, facile manipulation of their surface chemistry, adaptability in surface modifications, low toxicity, biocompatibility, biofilm inhibition, reactive oxygen species formation, shape and morphology controllable, and easy functionalization. Such intrinsic attributes pave the way for the tailored design and fabrication of MNPs, addressing specific challenges within the medical field [[305], [306], [307]].

The production and stabilization of MNPs can be achieved through various physical, biological, and chemical techniques, such as electrochemical manipulation, bio-enzymatic process, and photochemical reduction [308]. The choice of the synthesis method is critical as it significantly impacts the kinetics of the interaction between metal ions and the reducing agent, the adsorption process of the stabilizing agent on the MNPs, and therefore, the morphology, stability, and physicochemical characteristics of the resulting MNPs [309]. It is worth noting that MNPs can also naturally arise through abiotic and biotic processes that encompass erosion, combustion, precipitation, and even volcanic activities [310].

This interdisciplinary field harbors promising potential for creating new tools and strategies in biomedicine. Moreover, MNPs are postulated to play a crucial role in mitigating the development of MDR strains [311]. This potential is based on their ability to interact simultaneously with several biomolecules, which prevents the emergence of MDR strains, a severe concern in contemporary medicine [312]. A substantial cluster of research published in the scientific field has corroborated the MNPs antibacterial potential, implementing a plurality of mechanisms. The most notable includes the disruption and fragmentation of bacterial membranes and cell walls, inducing the expulsion of cellular content and a redox imbalance, triggering oxidative damage to the structure of bacterial DNA [313]. In MTB, the most prominent MNPs are ferromagnetic nanoparticles (Fe-MagNP), zinc oxide nanoparticles (ZnONPs), gold nanoparticles (AuNPs) and silver nanoparticles (AgNPs), which have demonstrated significant effectiveness against mycobacteria, both in *in vitro* bacterial culture models and at the intramacrophagic interface. Moreover, the most recent studies suggest that AgNPs could promote the susceptibility of MDR MTB strains, which generally show resistance to most organic antibiotics used in contemporary medicine [314,315].

In this context, Yu et al. [316] synthesized RIF-loaded polydopamine-functionalized silver nanoparticles (RIF@Ag-PDA NPs) targeting MDR-MTB strains. This combined nanosystem demonstrated a synergistic effect, enhancing antimycobacterial activity with a controlled release over 7 days. The RIF@Ag-PDA NPs exhibited a MIC of ≤1 μg/mL, in contrast to the standalone Ag-PDA NPs and free RIF with MIC of 128 and 64 μg/mL, respectively. Additionally, the nanosystem showed no cytotoxicity in mouse macrophage cell lines. This underscores the nanosystem potential in treating resistant TB infections. The study conducted by Zargarnezhad et al. [317] explored a methodology that combines Fe-MagNP with antibiotics to combat infections showing drug resistance. The authors developed a unique nanocomplex (INH-Fe-MagNP) that binds INH with Fe-MagNP (surface modified). This nanosystem was remarkably effective as an antibacterial agent, showing promising results against a variety of strains, including MTB*, Enterococcus faecalis*, *Staphylococcus aureus*, *Escherichia coli*, and *Pseudomonas aeruginosa*. The results compared the effectiveness of the nanosystem with free INH. In these assays, the MICs were 0.87, 1.26 and 62.52 μg/mL for INH-Fe-MagNP, free INH and Fe-MagNP, respectively. The observed reduction in INH concentration is attributed to the fact that INH is activated when it comes into contact with the KatG enzyme. INH-Fe-MagNP exhibits properties that mimic the enzymatic action, leading to a disruption in the MTB membrane, which accelerates this INH activation process. The implementation of these nanosystems have the potential to address INH-resistant MTB strains.

**Fig. 11:**
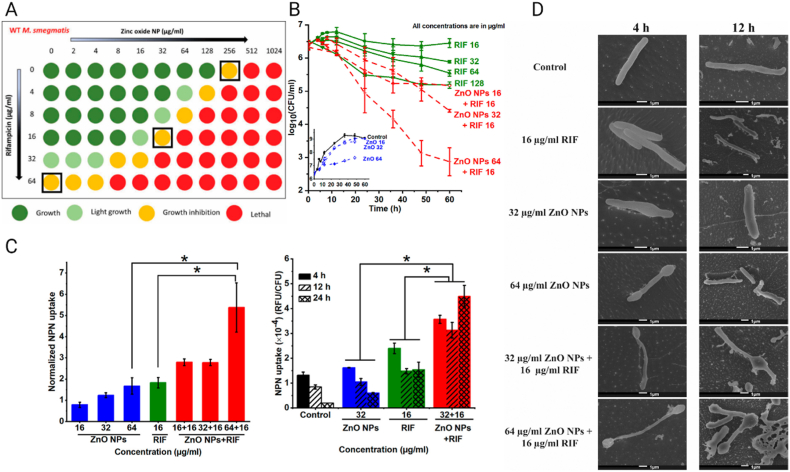
A) Depiction of the MIC through broth microdilution assay for the ZnO NPs and RIF combination against WT M. smegmatis at 37 °C over a period of 48 h. B) Kill kinetics of WT M. smegmatis when subjected to treatment with ZnO NPs and RIF. C) Examination of the WT M. smegmatis cells' morphology utilizing Cryo-SEM images following 4 and 12 h of ZnO NPs and RIF treatment. D) Impact of ZnO NPs and RIF on the membrane integrity of WT M. smegmatis cells. Reprinted/adapted with permission from Ref. [318]. Copyright 2020 American Chemical Society.

Mistry et al. [318] studied the synergistic action of ZnO NPs and RIF against *Mycobacteriym smegmatis*, a non-virulent strain like MTB. The ZnO NPs were synthesized through precipitation in organic media, using zinc acetate dihydrate and sodium hydroxide in methanol. The morphological characterization revealed a uniform distribution of the particles. The hydrodynamic average size was 11 nm, with a standard deviation of 2.72 nm. The zeta potential was +19.1 mV, indicating a positive surface charge. Additionally, UV–vis spectroscopy showed an absorption peak at 347 nm, confirming the formation of ZnO NPs. The ZnO NPs showed no inhibitory characteristics until reaching a concentration of 256 μg/mL; however, it was found that at 32 μg/mL it reduces the MIC of RIF from 64 to 16 μg/mL, indicating a synergistic interaction (Fig. 11A). In bacterial death kinetics experiments, the association of ZnO NPs and RIF was more effective in reducing the viability of *Mycobacterium smegnatis* compared to the free RIF. This decrease in viability was maintained up to 60 h, at which point no CFUs were detected (Fig. 11B). Drawing upon permeability assays and evaluations of mycobacterial membrane integrity, the authors suggest that the synergy between ZnO NPs and RIF generated significant damage to the bacterial membrane and an increase in RIF absorption at the intracellular level (Fig. 11C), which was corroborated by additional Cryo-SEM images (Fig. 11D). Furthermore, ZnO NPs were not harmful to THP-1 derived macrophages up to a concentration of 64 μg/mL. Building upon this promising outcome, ongoing efforts are dedicated to developing a targeted delivery system-like solid lipid nanoparticles or polymeric nanoparticles-loaded with both ZnO NPs and RIF. This system aims to effectively target bacteria within macrophages.

### 3.5 Mesoporous silica nanoparticles

Mesoporous silica nanoparticles (MSNs) were discovered in the latter stages of the 20th century [319] and their synthesis involve the use of self-assembling surfactants or co-polymers as templates for further condensation of silica precursors to achieve organic-inorganic hybrid structures. Following post-template removal, these structures showcase a distinctive inorganic porous network [320]. They possess a rich concentration of silanol groups, which promote functionalization, and a chemical composition like that of bioactive glasses. They are also marked by a systematic pore arrangement with size ranging from 2 to 50 nm, a significant pore volume, and a vast specific surface area [321,322].

The particle and pore structure are easily adjustable according to the choice of the synthesis parameters. These tunable nanosystems can load hydrophobic and hydrophilic molecules into the mesopores besides to be easily functionalized to design controlled and stimuli-responsive (*e.g*. pH, enzymes, ultrasound, and light) controlled drug delivery nanoplatforms for therapeutic purposes [[323], [324], [325]]. Thanks to their large surface area and high porosity, they can notably enhance interactions with microbial membranes. This could lead to oxidative stress in microorganisms and their destruction through physical contact and/or efficient release of antimicrobial agents [326]. In addition, MSN can enhance the immunomodulation of serum albumins targeting mitochondria in both *in vitro* and *in vivo* experiments. This activity is associated with the release of pro-inflammatory mediators, including tumor necrosis factor α, nitric oxide, and reactive oxygen species. These molecules play a direct role in the elimination of MTB [327]. The smart MSNs show high drug loading capacity, good biocompatibility, biodegradability, and clearance [328,329].

The focus on the nanometric diameter and versatility of the MSNs have led to the exploration of a wide range of applications in the biomedical field including cancer treatment [330,331], infectious diseases [332], bone diseases [333,334], etc.

INH undergoes an accelerated protonation process in low pH environments such as the GIT, which directly affects its bioavailability. This protonation action prevents adequate pharmacological absorption, reducing its therapeutic potential. This occurs because ionized INH shows a decrease in their lipid solubility and therefore an inability to efficiently cross the cell membrane, which can lead to the implementation of high daily dosage regimens to ensure therapeutic efficacy [335,336]. In this context, Almeida et al. [337] used MSNs to improve the bioavailability and provide INH controlled release. It was found that the INH-loaded MSNs was more effective at acidic pH due to the greater interaction between the silanol groups of the MSNs and the ionized INH. However, the INH release is more significant at neutral pH, especially in the intestine, due to the hydrogen interactions and electrostatic forces between the MSNs and INH. This process allows a prolonged and controlled INH release, which could maintain the therapeutic INH concentration in the bloodstream for a longer period, benefiting treatment effectiveness. The experimental data best fit the zero-order kinetic model, suggesting a slow and prolonged drug release. These results show that the use of pH-sensitive MSNs can improve the bioavailability of INH in GIT.

**Fig. 12:**
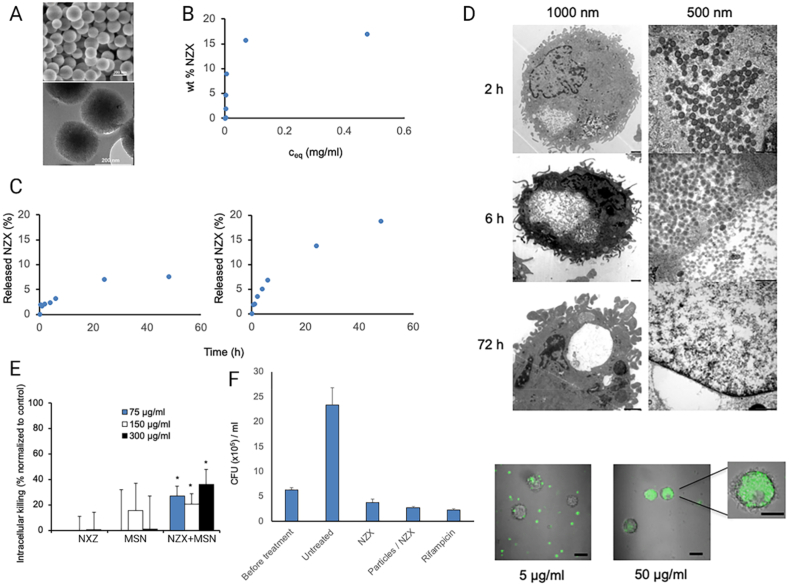
A) SEM image of bare MSN B) Absorption isotherm C) Release analysis of NZX-loaded MSN (PBS and SLF) D) CLSM of NZX@MSNs in macrophages E) Intracellular assay of NZX, MSN and NZX@MSN F) *In vivo* anti-MTB activity assay. Reprinted/adapted with permission from Ref. [338]. Copyright PLOS 2019.

Tenland et al. [338] reported the promising therapeutic potential of two mesoporous silica-based nanosystems composed of NZX and NZX combined with dipalmitoylphosphatidylcholine (DPPC) for TB treatment. According to electron microscopy images and nitrogen (N_2_) sorption analysis, the MSN exhibited an average diameter of 200 nm (Fig. 12A) and an average pore size of 3 nm, respectively. The N_2_ isotherm is very steep, indicating strong attractive interactions between the cationic NZX and the anionic MSNs (Fig. 12B). The release kinetics of NZX from MSN were studied in PBS and simulated lung fluid (SLF), showing a slow release in PBS (7.6 % at 48 h) and a faster release in SLF with DPPC (18.8 % at 48 h) (Fig. 12C). This indicates that the presence of DPPC in the SLF affects the NZX release, speeding it up under simulated pulmonary conditions. Confocal microscopy shows that the NZX@MSN were efficiently internalized by primary macrophages and stored within vesicle-like structures (Fig. 12D). In the framework of an intracellular infection model, it was found that NZX@MSNs were more effective at eradicating mycobacteria than the free NZX. Interestingly, at 75 μg/mL the NZX@MSNs exhibited greater activity against intramacrophagic MTB than at 150 μg/mL (Fig. 12E). An additional aspect of great relevance is that the MSNs did not trigger cytotoxicity in the macrophages, highlighting their potential as a safe nanovehicle for drug delivery. Additionally, *in vivo* tests were conducted using a murine model that was administered five doses of NZX@MSN or RIF via intratracheal route. The results indicated that the NZX@MSN achieved 88 % reduction in CFUs compared to the control group (untreated one), whereas free RIF accomplished a 90 % elimination, with no significant differences observed between the two treatments (Fig. 12F). These findings reinforce the potential of NDDS in combating latent TB.

Montalvo-Quirós et al. [339] evaluated the antimycobacterial activity of silver bromide nanoparticles-loaded MSNs (MSNs-AgNPs) and AgNPs-coated MSNs (Ag-MSNs). The MSNs-AgNPs was fabricated using one-pot method, followed by a thermal treatment to incorporate and reduce silver bromide into the MSNs framework. For the Ag-MSNs, CTAB and AgNO_3_ were mixed under controlled conditions and then, Na_2_BH_4_ was added as reducing agent. The resulting solution was left overnight to form a mesoporous silica layer around the AgNPs. The MSNs-AgNPs and Ag-MSNs exhibited hydrodynamic average size of 245.3 and 91.3 nm, respectively, with zeta potential of −19 mV for both nanosystems. Both nanosystems inhibited bacterial growth, however, MSNs-AgNPs stood out for their superior effectiveness, showing a MIC of 31.25 μg/mL, significantly lower than the 250 μg/mL exhibited by Ag-MSNs. Using the MSNs-AgNPs, a deterioration of the MTB bacterial membrane was observed, suggesting that its antimicrobial mechanism may be related to a direct interaction between the bacteria and the MSNs-AgNPs. In fact, both nanosystems represent a significant advantage because they allow the AgNPs uptake while preventing their aggregation in cells. The construction of a multifunctional nanosystem for the selective targeting and treatment of resistant TB strains would be possible associating the AgNPs-modified MSNs with the loading of drugs (*e.g*., antibiotics) inside the porous structure.

### 3.6 Polymeric nanoparticles

Polymeric nanoparticles (PNPs) have emerged in recent years as an interesting approach for efficient drug delivery [340]. These organic nanoplatforms have attracted attention due to their exceptional ability to uniformly integrate or encapsulate several compounds within a three-dimensional polymeric structure [341].

PNPs can present two fundamental designs depending on their architecture and morphology: nanospheres and nanocapsules. Nanospheres are defined by a polymeric matrix where the drug adheres, encapsulates, or dissolves, generating a monolithic or matrix system. In contrast, nanocapsules consist of a polymeric envelope that safeguards a liquid, aqueous or oily core, in which the drug dissolves [[342], [343], [344]].

The PNPs fabrication often requires the use of surfactants, organic amphiphilic molecules capable of self-assembling in solution, which act as stabilizers during nanoemulsion, facilitating the generation of a well-defined nanoparticle structure [345]. Additionally, surfactants can decrease the surface tension of nanoparticles, increase affinity with lipid structures and even reduce the average diameter of nanoparticles, also acting as cryoprotective agents [346]. PNPs are well-known for their robust stability and potential for large-scale production. The natural polymers such as albumin, hyaluronic acid, dextrose, collagen, and chitosan, as well as synthetic polymers, such as poly DL-lactic-co-glycolic acid (PLGA) and polyethylene glycol have been using on the production of these nanosystems [347,348].

The scope of PNPs applications is vast, extending beyond the pharmaceutical sector to encompass areas such as photonics, electronics, environmental and biotechnology [349,350]. Additionally, PNPs enhance the bioavailability and solubility of therapeutic compounds, allowing for the administration of drugs with low water solubility in smaller doses, which reduces side effects and ensures precise dosing. This extends the retention time, facilitates controlled and sustained release, and improve patient compliance [351,352]. Furthermore, PNPs exhibit no toxic, inflammatory, or immunogenic effects [353,354]. These nanoparticles can accurately carry drugs, proteins or genetic material to specific organs or cells [355,356]. In addition, their stability in the GIT allows them to protect the encapsulated compounds from pH fluctuations and enzymatic degradation [357], making them interesting nanosystems to fight against TB.

Recently, Primo et al. [358] described the development of PNP-loaded RIF functionalized with an antimicrobial peptide (labeled Ctx (Ile)21-Ha), utilizing the ionic gelation method. The peptide was attached to the surface of chitosan that was pre-functionalized with N-acetylcysteine; the free SH group of the N-acetylcysteine formed a disulfide bridge with the terminal cysteine of the peptide, creating a peptide corona around the nanoparticles, termed Ctx-NAC-CSNP. The nanosystems, the peptide, and free RIF were evaluated both in sensitive strains and clinical isolates of MTB. The hydrodynamic average size ranged from 123.9 to 248.9 nm, with a zeta potential from +29 to +39 mV. The MIC of free RIF was approximately 0.977 μg/mL for sensitive strains, but it showed no activity against clinical isolates; the MIC of the free peptide was 14 μg/mL. The Ctx-NAC-CSNP showed no antimicrobial activity; however, RIF-encapsulated Ctx-NAC-CSNP exhibited an MIC of approximately 0.977 μg/mL in both sensitive strains and clinical isolates resistant to RIF, without showing cytotoxicity. Confocal microscopy revealed significant penetration of the nanosystems into macrophages compared to free RIF. These findings highlighted the system's potential to sensitize MDR and XDR MTB strains, suggesting a viable alternative to long-term treatments for infections involving intracellular bacterial growth.

**Fig. 13:**
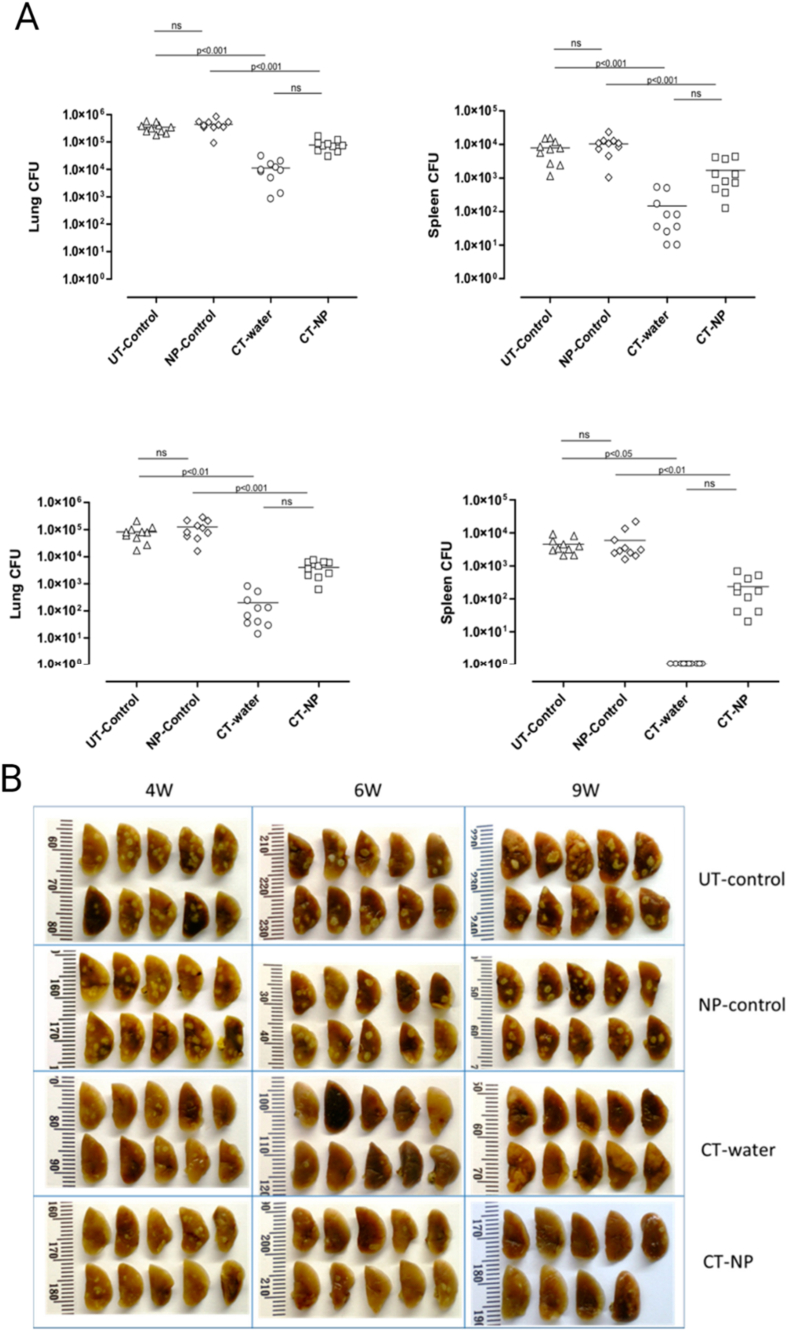
A) Kinetics of the bactericidal efficacy of the treatment applied to the lungs and spleen of mice and B) the pulmonary pathology in the infected animals throughout the experimental period [359]. Reprint published from an open access article by MDPI.

Aiming to decrease the treatment frequency for TB, Kalombo et al. [359] formulated and assessed the efficacy of PLGA PNPs loaded with INH, RIF, and PZA, utilizing a murine model for an aerosol inhalation challenge test. The nanosystems were designed using a double emulsion spray drying technique incorporating polyethylene glycol and polyvinyl alcohol. The formulations exhibited average size up to 400 nm with a polydispersity index (PDI) ranging from 0.2 to 0.4, and a zeta potential between +17 and + 20 mV. For the *in vivo* evaluation, the authors employed a mouse model infected with MTB H37Rv. Following three weeks of infection, the mice were administered either a daily oral dose of the free-form drugs for nine weeks or a single weekly dose of the drug-loaded PLGA PNPs. By the fourth week of treatment, there was a notable reduction in the bacilli load in both groups (empty nanoparticles and untreated mice) relative to the control groups, observed in both the lungs and spleen (Fig. 13A). As expected, the untreated groups (UT-control) and the empty particles (NP-control) exhibited a significant number of pulmonary lesions. The groups that received chemotherapy in water (CT-water) and the encapsulated particles chemotherapy (CT-NP) demonstrated a marked reduction in lesions, with the CT-NP group achieving the best outcomes (Fig. 13B). These nanoparticles have the potential to release the drug in a sustained manner, which could lead to fewer doses and enhanced patient adherence to the therapeutic regimen. A metered release lessens the body's exposure to the drug, mitigating potential toxic side effects. The encapsulation technique also acts as a protective shield for the pharmaceutical agent against the acidic stomach environment, essential for substances like RIF that are sensitive to extremely low pH levels.

Mukhtar et al. [360] focused on effective drug delivery to affected lung tissue, developing a dry powder for inhalation with the ability to avoid the accumulation of drugs outside the action site. They used a nano-hybrid approach, using non-toxic and biodegradable polymers like chitosan and hyaluronic acid, to manufacture the INH-loaded PNPs through the ionic gelation method. In addition, the polymer was mannosylated to improve uptake by macrophages. The results demonstrated that the nanosystems presented high encapsulation efficiency (92.31 % INH-MC/HA NPs and 92.18 % INH-CS/HA NPs), excellent stability with a positive zeta-potential (+34.3 and + 29.5 mV, respectively), which contributes to intracellular absorption as observed through CLSM. The hydrodynamic average particles size (303 and 342 nm, respectively) are within the optimal range for deposition in the alveolar region of the lungs. In addition, cytotoxicity assays confirmed the safety of INH-MC/HA NPs for human cells.

**Fig. 14:**
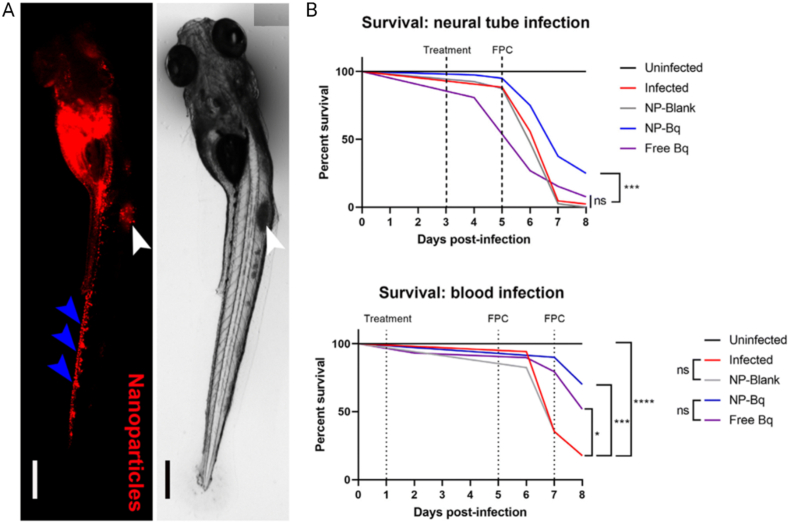
A) Accumulation of nanoparticles in tubal neural granulomas and B) Survival analysis comparing the therapeutic efficacy of free BQ versus NP-BQ. Reprinted/adapted with permission from Ref. [361]. Copyright Royal Society of Chemistry 2023.

Similarly, Bhandari et al. [361] implemented the use of *Danio rerio*, commonly known as zebrafish, to explore the biodistribution and biological interactions of BQ-loaded polymeric micellar nanoparticles (NP-BQ), within the context of MTB infection. The authors used high-resolution *in vivo* imaging techniques to assess efficacy of the treatment. The micelles were synthesized by dissolving the polymer in DMSO and adding phosphate buffer with a syringe pump at flow rate of 0.1 mL/min. The resulting solution was subjected to dialysis against water, followed by lyophilization to obtain the micelles. The hydrodynamic average size of the nanoparticles ranged from 40 to 60 nm, showing good polydispersity index (PDI) of up to 0.2. A neural tube infection model in zebrafish was used, which results in the formation of a large and localized granuloma, as opposed to blood inoculation that generates dispersed bacterial foci. Mycobacteria were inoculated 3 days post-fertilization, followed by the intravenous administration of 8.3 ng of NP-BQ 24 h later. Through a fluorescence stereomicroscope, a significant accumulation of NP-BQ in the neural tube granulomas was detected (Fig. 14A). Approximately 3.4 % of the administered NP-BQ was located in the granuloma 24 h after injection, an amount almost five times greater than the 0.75 % detected in a non-infected control area adjacent to the granuloma. Additionally, for the survival experiments, a dose of 20 nL per embryos (0.415 mg/mL) was injected, in its encapsulated or free form. The NP-BQ showed a 10 % increase in survival compared to the free BQ. The results of fluorescence pixel counting (FPC) showed that NP-BQ increased post-treatment survival and decreased bacterial load in MTB-infected zebrafish embryos compared to the control group (NP-Blank) and the free BQ (Fig. 14B), in addition to reducing drug-related toxicity. The authors propose this innovative solution to address the complexities of TB treatment, enhancing drug distribution, reducing toxicity, and increasing treatment efficacy. These advancements represent a significant step towards overcoming the current barriers in the fight against this devastating disease, paving the way for the development of more effective and safer therapies.

## 4 Gap until clinical study

The promise of nanotherapeutics in the fight against TB has sparked considerable interest within the research community. Nanoparticles stand out for their ability to enhance drug delivery by targeting TB with greater precision, improving drug stability, and enabling sustained release. These benefits could address the limitations of traditional TB treatments, such as poor drug penetration into infected tissues, the need for frequent dosing, and the rise of drug-resistant TB strains. Researchers have extensively studied nanocarriers including liposomes, polymeric nanoparticles, and lipid-based nanoparticles, for effective anti-TB drug delivery. Preclinical studies have shown promising results, including improved drug effectiveness, lower toxicity, and increased antimicrobial activity against MTB [250,[362], [363], [364], [365], [366], [367]]. Additionally, nanocarriers can load multiple drugs and promote simultaneous drugs delivery [[368], [369], [370]].

The clinical application of nanomedicines for TB remains in the initial stages, with research focusing on evaluating their safety, efficacy, and pharmacokinetics in humans. Clinical trials are crucial to validate the potential of nanomedicines, optimizing treatment approaches, and addressing production, scalability, and regulatory challenges. A detailed review of clinical trials registered on https://clinicaltrials.gov/as of March 11, 2023, using specific search terms like “nano”, “nanosystems”, “nanoparticle”, “liposome”, and “nanotechnology”, while excluding “vaccines” and “diagnostic markers” to concentrate on therapeutic applications, identified no completed studies on nanomedicines application against MTB. This gap highlights the untapped potential for innovative nanoparticles-based treatments for active and latent MTB infections. Although the initial skepticism towards nanoparticle-based therapies, the approval of several drugs-loaded nanoparticles by the FDA underscores the viability and potential of this approach (Table 4).

**Table 4:** FDA-approved drugs-loaded nanosystems to treat various diseases.

NanoDrug	Specifications	References
Onivyde (liposomal irinotecan)	This medication is a liposomal form of irinotecan, sanctioned for treating metastatic pancreatic cancer along with other chemotherapy drugs.	[371]
Myocet (liposomal doxorubicin)	Myocet is another variant of doxorubicin enclosed in liposomes, given the green light for managing metastatic breast cancer.	[372]
Depo-Provera (medroxyprogesterone acetate)	Depo-Provera comprises medroxyprogesterone acetate encapsulated within polymer microspheres, endorsed as a long-acting injectable contraceptive.	[373]
Triamcinolone acetonide injectable suspension (Kenalog)	Kenalog consists of triamcinolone acetonide suspended in nanometer-sized particles, approved to alleviate various inflammatory conditions like arthritis, dermatitis, and allergy.	[374]
Doxil (liposomal doxorubicin)	Doxil is a liposome-packaged form of doxorubicin, authorized for treating ovarian cancer, Kaposi's sarcoma, and multiple myeloma.	[375]
AmBisome (liposomal amphotericin B)	This medication is a liposome-encased variant of amphotericin B, sanctioned for addressing systemic fungal infections such as candidiasis and aspergillosis.	[376]
Nanoxel (nanoparticulate paclitaxel)	Nanoxel is a nanoparticle configuration of paclitaxel, permitted for managing diverse cancer types like breast cancer, ovarian cancer, and non-small cell lung cancer.	[377]
Vyxeos (liposomal daunorubicin and cytarabine)	Vyxeos is a liposomal formulation combining daunorubicin and cytarabine, authorized for treating acute myeloid leukemia in adults.	[378]
Marqibo (liposomal vincristine)	Marqibo is a liposomal version of vincristine, approved for treating acute lymphoblastic leukemia in adult patients.	[379]
DepoDur (liposomal morphine sulfate)	DepoDur is a liposomal formulation of morphine, endorsed for managing postoperative pain.	[380]
Lipodox (liposomal pegylated doxorubicin)	Lipodox is a liposomal variant of pegylated doxorubicin, permitted for treating ovarian cancer and metastatic breast cancer.	[381]
Diprivan (Propofol Lipuro)	Diprivan is a propofol emulsion utilized as a general anesthetic. Propofol is encapsulated within a lipid formulation, enhancing its solubility and bioavailability. While not specifically tailored for pulmonary treatment, it is commonly administered during pulmonary surgical procedures to induce and sustain anesthesia.	[382]
Abraxane (albumin-bound paclitaxel)	Abraxane is a nanoparticle formulation of albumin-bound paclitaxel, cleared for treating various cancer types such as breast, lung, and pancreatic cancer.	[383]

The clinical potential of nanomedicines in combating MTB marks an exciting frontier for innovation in TB treatment. Sustained research and clinical development are essential to fully harness the therapeutic advantages of the smart drug delivery nanosystems and ultimately enhance outcomes for TB patients globally.

## 5 Conclusion and future perspectives

The conventional treatment for TB confronts significant challenges across multiple dimensions, including pharmaceutical issues related to drug toxicity, stability, biodistribution, and bioavailability; biological hurdles arising from the nature of mycobacteria, resistance, and the continual evolution of MTB. Additionally, the elevated costs of medications in underdeveloped countries result in considerably higher infection rates, underscoring the economic barrier as a critical concern in these regions. NDDS have emerged as an innovative and efficient therapeutic approach for TB, aiming to surmount these barriers and achieve global accessibility. NDDS are notable for their ability to enhance the biodistribution of drugs, with a specific focus on lung tissues, ensuring effective penetration in both macrophages and granulomas. They provide controlled drug release at therapeutic levels and maintain plasma stability. These systems not only have the potential to sensitize MDR and XDR TB strains but also to improve the solubility of encapsulated drugs, ensuring precise dosing. Such accuracy is crucial for mitigating adverse effects such as hepatotoxicity, hyperuricemia associated with PZA, EMB-related ocular toxicity, and INH-induced peripheral neuropathy. Additionally, NDDS offer substantial protection for drugs against detrimental factors like pH fluctuations and enzymatic degradation, thus maximizing their efficacy. This characteristic is particularly beneficial for the administration of drugs susceptible to proteases and peptidases. The foremost advantage of NDDS lies in their potential to strengthen patient adherence to treatment by simplifying dosing regimens, which contributes to treatment completion. Furthermore, NDDS provide flexibility in administration routes, ranging from inhalation delivery targeting the lungs directly to oral and intravenous methods. Despite promising initial findings, it is imperative to deepen research and meticulously assess potential long-term side effects, with progress to preclinical and clinical trials being essential. The scientific literature indicates a paucity of *in vivo* studies that utilize NDDS for TB treatment. Dartois and Rubin [384] have reported that only two drugs are in the preclinical phase, four in clinical phase I, and six covering clinical phases II and III targeted at TB, with none employing nanosystems for delivery. Therefore, continuing research advances in this domain is vital given the numerous benefits outlined previously, particularly targeted delivery to the site of infection. Our literature review has determined that drug encapsulation can also significantly lower the minimum inhibitory concentration necessary to eradicate MTB, potentially allowing for the use of reduced drug amounts, thereby generating economic savings for patients.

Future research should aim for a deeper understanding of the mechanisms through which NDDS improve the effectiveness of treatment against MDR and XDR TB strains, and how they can be optimized to maximize their efficacy. Further research is required to assess the feasibility of large-scale production and scalability of these nanomedicines, ensuring they can be an accessible and affordable option for patients worldwide. It is worth emphasizing that further studies towards inhalation administration route are essential to enhance the treatment's efficacy directly in the lungs. Evaluating the long-term impacts of NDDS on the human body also represents a critical area that needs great attention from researchers, including deeper studies regarding the potential accumulation in specific organs. Finally, in the development and application of nanomedicines, profound ethical issues emerge that intertwine with safety and legal responsibility, highlighting the urgency for thoughtful and proactive governance in this scientific domain. The proliferation of nanomaterials as drug delivery systems not only promises radical transformations in nanomedicine, offering more precise and less invasive treatments, but also entails potential risks whose magnitude and nature are not yet fully understood. This context demands a robust and adaptable regulatory framework, capable of balancing the promise of innovation with the necessary caution to protect public health and the environment. The ethical implications of nanomedicines range from equity in access to innovations to privacy and consent in the era of personalized medicine, challenging lawmakers to create policies that not only address tangible risks but also anticipate the far-reaching social and ethical consequences of these technologies. In this context, international collaboration and the harmonization of standards become imperative to forge a global governance that respects cultural and socioeconomic differences, while promoting a fair exchange of knowledge and benefits.

## Ethics approval and consent to participate

Not applicable. No clinical study, animal experiments, or human subjects.

## CRediT authorship contribution statement

**Christian S. Carnero Canales:** Writing – review & editing, Writing – original draft, Validation, Investigation, Conceptualization. **Jessica Ingrid Marquez Cazorla:** Writing – review & editing, Writing – original draft, Visualization, Validation, Investigation, Formal analysis. **Renzo Marianito Marquez Cazorla:** Writing – review & editing, Writing – original draft, Visualization, Validation, Methodology, Investigation, Formal analysis. **Cesar Augusto Roque-Borda:** Writing – review & editing, Visualization, Validation, Supervision. **Giulia Polinário:** Writing – review & editing, Writing – original draft, Investigation, Formal analysis. **Rufo A. Figueroa Banda:** Writing – review & editing, Writing – original draft, Investigation, Formal analysis. **Rafael Miguel Sábio:** Writing – review & editing, Writing – original draft, Visualization, Validation, Investigation, Formal analysis, Conceptualization. **Marlus Chorilli:** Writing – review & editing, Validation, Supervision, Conceptualization. **Hélder A. Santos:** Writing – review & editing, Validation, Supervision, Resources, Project administration, Conceptualization. **Fernando Rogério Pavan:** Writing – review & editing, Visualization, Validation, Supervision, Resources, Project administration, Funding acquisition, Conceptualization.

## Declaration of competing interest

All contributing authors declare no conflict of interest.
